# Supplementary material for: Paneth cell SIRT1 deficiency increases intestinal stress resistance by modulating the gut microbiota
Source: EMBO Rep. 2026 Mar 13;27(7):1830–57. doi: 10.1038/s44319-026-00726-3 (PMC13076647; doi:10.1038/s44319-026-00726-3)
Supplement: Supplementary file 1 — Appendix [file 44319_2026_726_MOESM1_ESM.pdf]

Appendix for 'Paneth cell SIRT1 deficiency increases intestinal stress resistance by modulating the gut microbiota'

Table of Contents

Appendix Figure S1 -- Page 2

Appendix Figure S2 -- Page 3

Appendix Figure S3 -- Page 4

Appendix Figure S4 -- Page 5

Appendix Table S1 -- Page 6

Appendix Table S2 -- Page 7

Appendix Table S3 -- Page 8

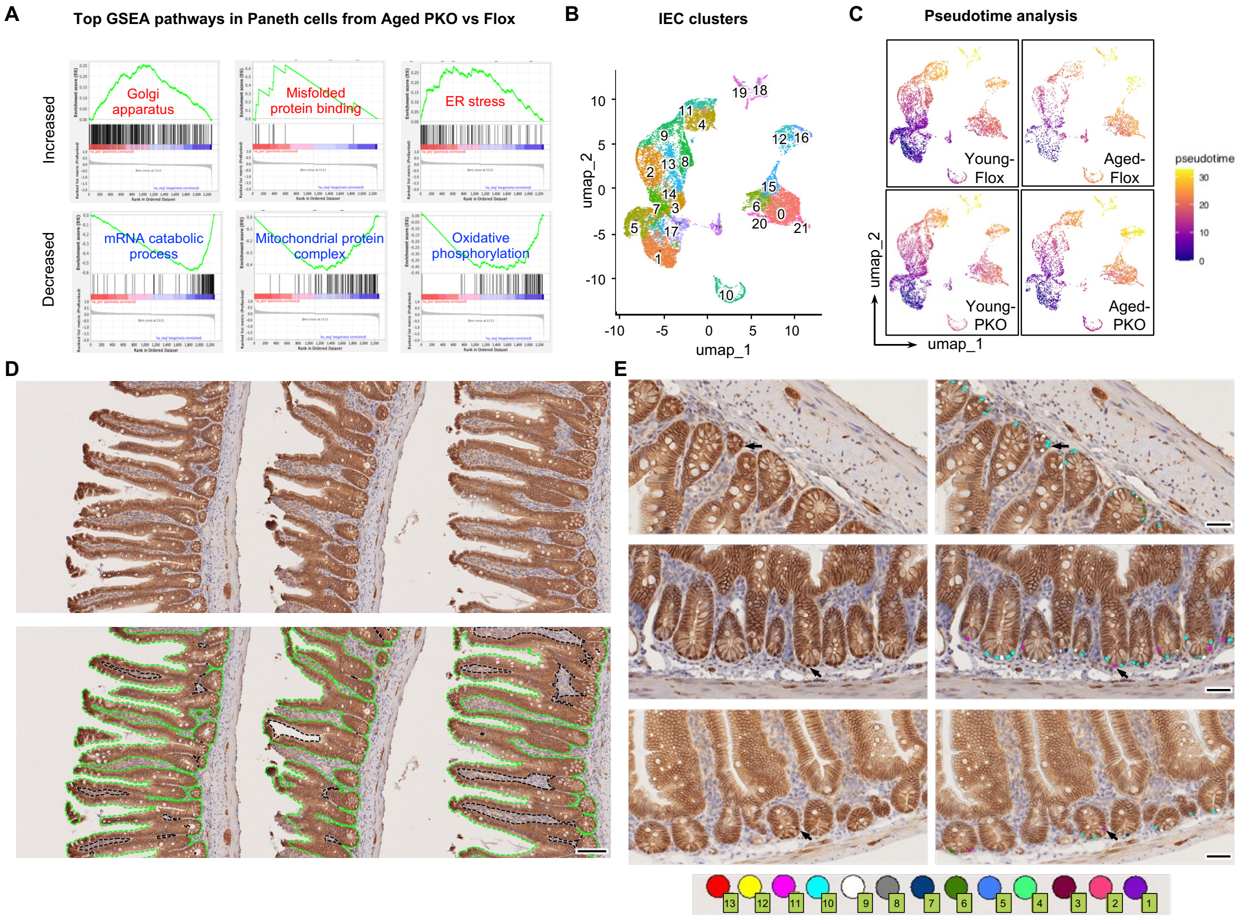

Appendix Figure S1. SIRT1 deficiency in Paneth cells cell-autonomously induces ER stress response and enhances Wnt signaling.

(A) SIRT1 KO Paneth cells have increased response to ER stress but with defective mitochondrial activities. Transcriptomes of Flox and SIRT1 KO Paneth cells from scRNA-seq dataset were analyzed. Top enriched Gene set enrichment (GSEA) pathways in SIRT1 KO vs Flox Paneth cells in aged mice are shown. (B) Cell clusters of IECs in the small intestine of Flox and SIRT1 PKO mice (C) Pseudotime analysis of IECs in the small intestine of young and aged Flox and SIRT1 PKO mice. (D-E) AI-assisted image analysis of nuclear β-catenin staining intensity. (D) Small intestine, IHC for β-catenin, detected epithelial tissue. (Top) Original image at approximately 10x magnification. (Bottom) Automatically detected epithelial tissue is outlined by green and black dotted lines. Bar, 100 μm. (E) Small intestine, IHC for β-catenin detected and scored nuclear localization. (Left) Original images at approximately 40x magnification. (Right) Segmented nuclei color coded by automatically determined intensity score, with color legend on the bottom. Groups from top to bottom: Aged Flox, Aged PKO, Young Flox, Young PKO. Arrows showing brown stained nuclei. Bars, 50 μm.

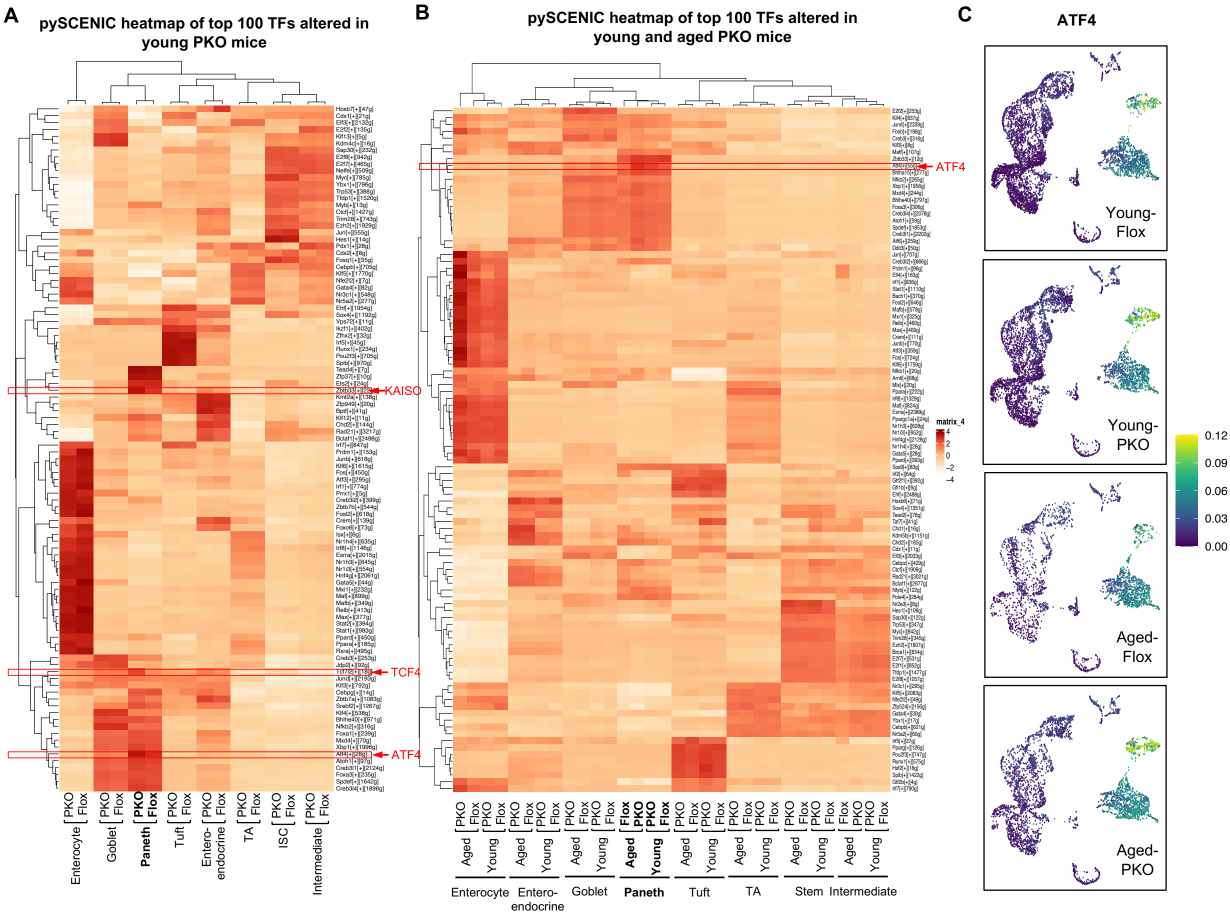

Appendix Figure S2. SIRT1 deficiency in Paneth cells cell-autonomously enhances Wnt and ATF4 signaling.

(A) pySCENIC heatmap of top 100 TFs altered in young PKO mice. (B) pySCENIC heatmap of top 100 TFs altered in young and aged PKO mice. (C) The transcription activities of ATF4 in small intestinal epithelial cells in young and aged mice. Cell clusters from young and aged Flox and SIRT1 PKO mice were analyzed by scRNA-seq. All the combined promoters of the genes expressed in a cell were used to infer transcription factor activity by pySCENIC, and the activity of ATF4 in different epithelial cells was projected onto tSNE space with their activity color coded.

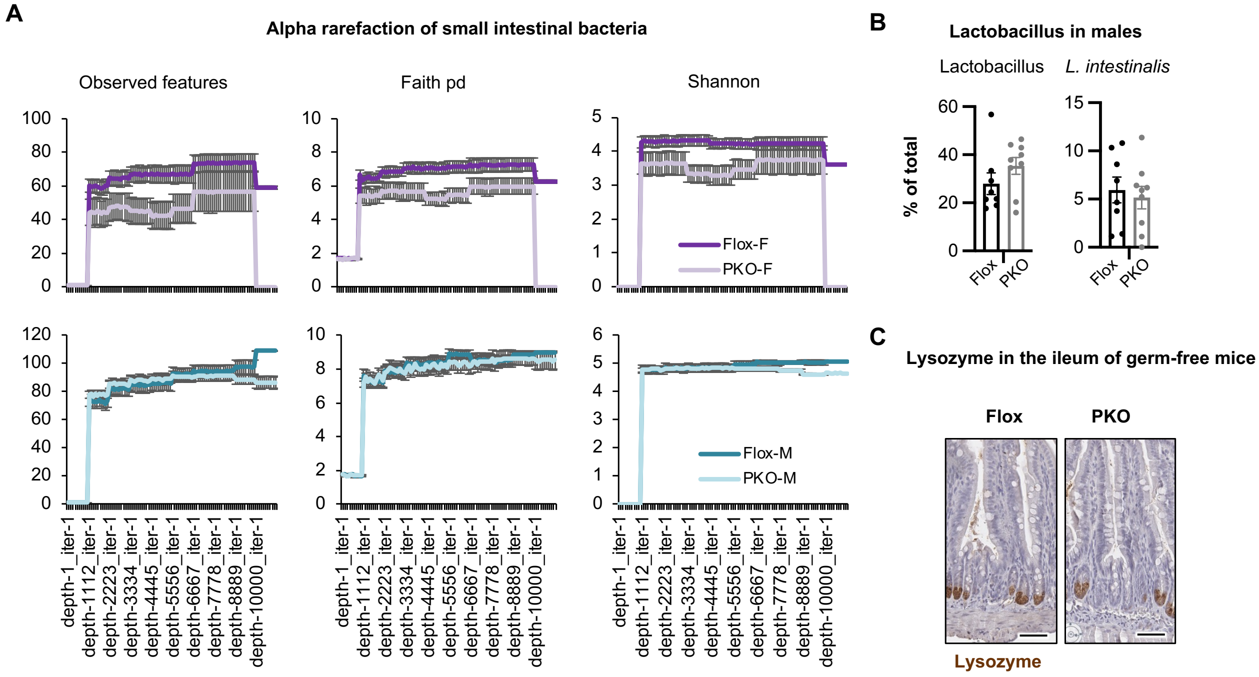

Appendix Figure S3. Paneth cell SIRT1 modulates Paneth cells and small intestinal microbiota.

(A) Alpha rarefraction of small intestinal bacteria. Total small intestinal DNA from Flox and SIRT1 PKO mice were analyzed for mucosal adherent microbiota using 16S rRNA gene amplicon sequencing as described in Methods (n=10 Flox and 8 PKO females and 10 Flox and 10 PKO males). (B) The abundance of Lactobacillus in the small intestine of male Flox and PKO male mice. The mucosal adherent microbiota in the small intestine of male mice were analyzed as in (A) (n=10 Flox and 8 PKO females and 10 Flox and 10 PKO males, values are expressed as mean ± SEM; Student's t-test, not significant). (C) Depletion of microbiota diminishes Lysozyme+ Paneth cells in both Flox and PKO mice. Bars. 50 μm.

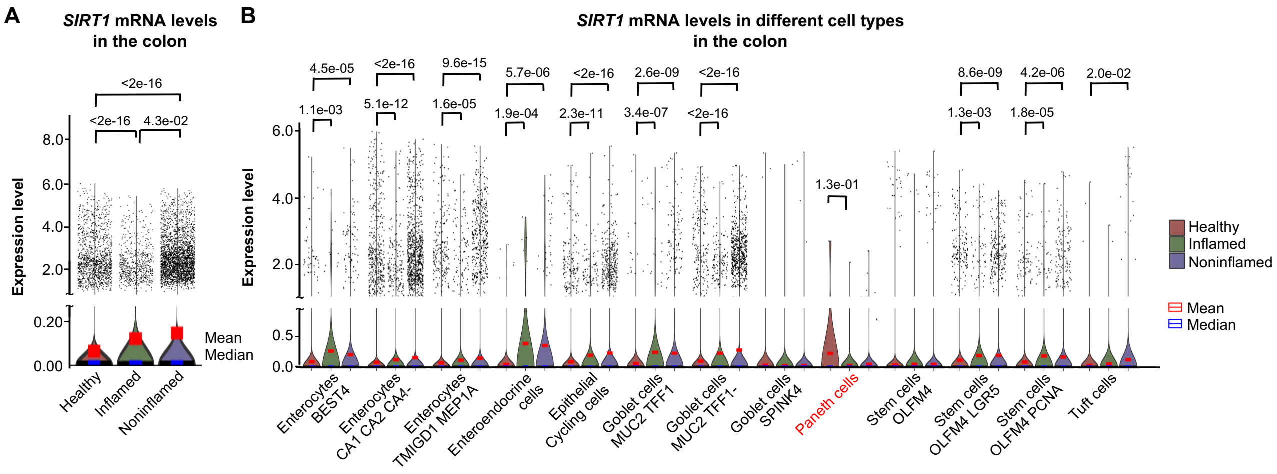

Appendix Figure S4. scRNA-seq analysis of intestinal epithelial cells isolated from the colon of healthy donors or CD patients.

scRNA-seq datasets (Accession DUOS-000146 CD_Atlas_2021_GIDER; DUOS-000145 CD_Atlas_2021_PRISM) of intestinal epithelial cells from healthy donors or CD patients was analyzed. (A) The expression levels of SIRT1 in the colon were compared between Healthy controls, inflamed, and non-inflamed samples. (B) The mRNA levels of SIRT1 in different cell types in the colon. For both (A) and (B), two-sided Wilcoxon test, and adjusted p-values were obtained using the Holm–Bonferroni correction.

Cell type	Cluster	Top markers	Cell number				% of total cells			
			Young-Flox	Young-PKO	Aged-Flox	Aged-PKO	Young-Flox %	Young-PKO %	Aged-Flox %	Aged-PKO %
Lgr5+ stem cell	1	*olfm4, Ifitm3, Jam1, Slc12a2, Clca3b, Lgr5*	738	544	169	413	12.62%	9.94%	6.05%	9.22%
	5	*olfm4, Stmn1. Ifitm3, Top2a, Tubb5, Lgr5*	467	329	108	218	7.98%	6.01%	3.86%	4.87%
Intermediate (Lgr5-)	3	*Ube2c, Pclaf, Top2a, Birc5, Cdk1*	571	474	113	165	9.76%	8.66%	4.04%	3.68%
	7	*Dmbt1, Hspd1, Snghg4, Mcm6, Clqbp*	298	233	112	201	5.09%	4.26%	4.01%	4.49%
	14	*Pclaf, Tuba1b, Ube2c, Reg3g, Reg3b*	121	303	40	107	2.07%	5.54%	1.43%	2.39%
	17	*Hmmr, Cps1, Rpgrip1, Mik67, Hnrnpa1*	183	110	107	118	3.13%	2.01%	3.83%	2.63%
TA cell	2	*Dmbt1, Rbp1, Gpx1, Reg1, Arg2, Krt19*	497	395	210	305	8.50%	7.22%	7.51%	6.81%
	8	*Fabp6, Mgat4c, Reg3b, Reg3g, Gsdmc4*	340	306	74	113	5.81%	5.59%	2.65%	2.52%
	9	*Reg1, Adh6a, Gsta1, Sis, Il18, Reg3a, Arg2*	270	258	159	134	4.62%	4.71%	5.69%	2.99%
	13	*Reg3g, Gsdmc4, Car4, Bcl2l15, Gsdmc2, Krt19*	151	251	51	183	2.58%	4.59%	1.82%	4.09%
Enterocyte	4	*Fabp1, Apoc*	315	493	110	261	5.38%	9.01%	3.94%	5.83%
	11	*Fabp6, Fabp2, Apoa1,*	216	165	71	200	3.69%	3.01%	2.54%	4.47%
Goblet	0	*Clca1, Agr2, Tff3, Muc2, Atoh1,*	555	516	515	606	9.49%	9.43%	18.43%	13.53%
	6	*Agr2, Selenom, Ccl9, Atoh1, Tff3, Klf4*	229	182	205	238	3.91%	3.32%	7.33%	5.31%
	15	*Rnase1, Ccl9, Mmp7, Ang, Atoh1, Muc2*	128	110	120	166	2.19%	2.01%	4.29%	3.71%
	20	*Wfdc18, Tff2, Muc2, Tff3*	108	64	83	67	1.85%	1.17%	2.97%	1.50%
	21	*Zg16, S100a6, Ido1, Clca1, Muc2, Tff3*	73	52	37	45	1.25%	0.95%	1.32%	1.00%
Paneth	12	*Defa30, Defa23, Clps, Defa24, Defa17, Lyz1, Ang4*	127	187	107	216	2.17%	3.42%	3.83%	4.82%
	16	*Defa22, Defa21, Defa5, Defa34, Defa36, Ang4, Lyz1, Wnt3*	118	161	15	226	2.02%	2.94%	0.54%	5.05%
Endocrine	18	*Gip, Sst, Gcg, Nts, Cck, Pyy, Sct, Ghrl*	83	100	87	111	1.42%	1.83%	3.11%	2.48%
	19	*Chgb, Chga, Tac1, Reg4, Ghr1. Tph1, Cim*	81	91	81	128	1.38%	1.66%	2.90%	2.86%
Tuft	10	*Rgs13, Lrmp, Hck, Alox5ap, Kctd12, Avil, Espn*	181	150	221	258	3.09%	2.74%	7.91%	5.76%
Total			5850	5474	2795	4479	100.00%	100.00%	100.00%	100.00%

Appendix Table S1. Cell clusters of total intestinal epithelial cells (IEC) from female Flox and PKO mice.
Total live IECs from scRNA-seq dataset were annotated as described in Methods.

TF name	Gene Number	Gene name
Atf4(+)	55	*Vcpip1,Ube2w,Gmppa,Rhbdd1,Hdlbp,Ppp1r15b,Zbtb18,Hspa5,Ttc17,Chac1,Eif2s2,Zfp335,Slc7a11,Siah2,Ints3,Tmem56,Dcaf10,Focad,Insig1,Rell1,Rchy1,Asns,Prr14,Bag3,Chid1,Dusp8,Evi5l,3930402G23Rik,Msmo1,Klf2,Herpud1,Aars,Mon1b,Herpud2,Tmem30a,Nus1,Sar1a,Aldh1l2,Mon2,Mars,Rab1a,Psme4,Alkbh5,Sp2,P4hb,Dnajb9,Sel1l,Larp4b,Srebf2,Slc38a2,Ltn1,Gnmt,Osbp,Usp9x,Magt1*
Zbtb33(+)	12	*Nr4a2,Rnf32,Tubgcp5,Nars2,Mgmt,Rasa3,Mmp7,Ccdc92b,Smarcd1,Ppp2r2b,Neto1,Habp2*

Appendix Table S2. Significantly changed ATF4 and KAISO target genes in SIRT1 KO Paneth cells.

Transcriptomes of Paneth cells from SIRT1 PKO and Flox mice in scRNA-seq dataset were analyzed using pySCENIC as described in Methods. The ATF4 and KAISO target genes that are significantly changed in Paneth cells from SIRT1 PKO vs Flox mice were identified.

Criteria	Grade	Description
**Inflammation**	None (0)	Absence of inflammatory cells
	Minimal (1)	Few inflammatory cells in clusters in the mucosa
	Mild (2)	Few inflammatory cells in the mucosa and with a patchy distribution
	Moderate (3)	Moderate numbers of inflammatory cells in the mucosa and submucosa and occasionally into muscularis layers with a patchy distribution
	Marked (4)	Numerous inflammatory cells diffusely distributed in the mucosa, submucosa and frequently extending into the muscularis layers and beyond to the serosa
**Ulceration**	None (0)	Absent
	Minimal (1)	Loss of 1-25% of the mucosa
	Mild (2)	Loss of 26-50% of the mucosa
	Moderate (3)	Loss of 51-75% of the mucosa
	Marked (4)	Loss of 76-100% of the mucosa
**Edema**	None (0)	Absence of edema
	Minimal (1)	Minimal expansion of submucosa
	Mild (2)	Mild expansion of submucosa
	Moderate (3)	Moderate expansion of submucosa and sometimes extending to the muscularis layers
	Marked (4)	Massive expansion of submucosa and muscularis layers
**Goblet cell loss**	None (0)	Absent
	Minimal (1)	Minimal loss of goblet cells involving 1-25% of mucosa
	Mild (2)	Mild loss of goblet cells involving 26-50% of mucosa
	Moderate (3)	Moderate loss of goblet cells involving 51-75% of mucosa
	Marked (4)	Marked loss of goblet cells involving 76-100% of mucosa
**Granulation tissue**	None (0)	Absence of granulation tissue
	Minimal (1)	Minimal granulation tissue associated with the ulceration site(s) and focally distributed
	Mild (2)	Mild granulation tissue associated with ulceration site(s) with a patchy distribution
	Moderate (3)	Moderate, focally extensive granulation tissue associated with ulceration site(s)
	Marked (4)	Extensive granulation tissue associated with ulceration site(s) and diffusely distributed

Appendix Table S3. Histopathological grading criteria of the colon of DSS-treated mice.
