# Supplementary figures and images for: Paneth cell SIRT1 deficiency increases intestinal stress resistance by modulating the gut microbiota
Source: EMBO Rep. 2026 Mar 13;27(7):1830–57. doi: 10.1038/s44319-026-00726-3 (PMC13076647; doi:10.1038/s44319-026-00726-3)

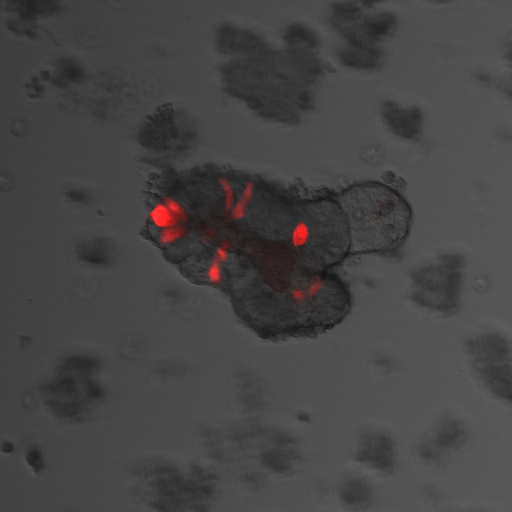

Supplement: Supplementary file 10 — Source data Fig. 1 [file 44319_2026_726_MOESM10_ESM.zip › Figure 1/Figure 1D-PHet 3.tif]

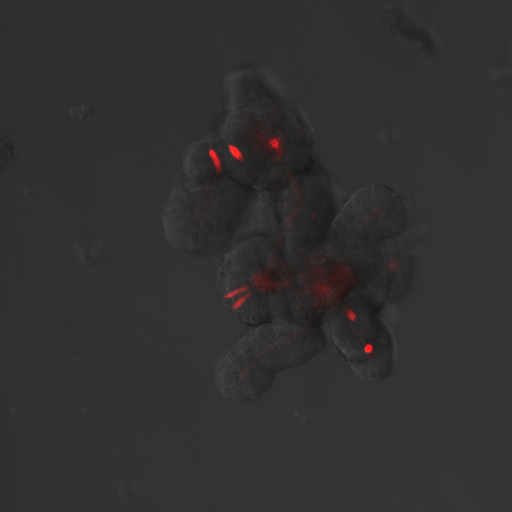

Supplement: Supplementary file 10 — Source data Fig. 1 [file 44319_2026_726_MOESM10_ESM.zip › Figure 1/Figure 1D-PHet 1.tif]

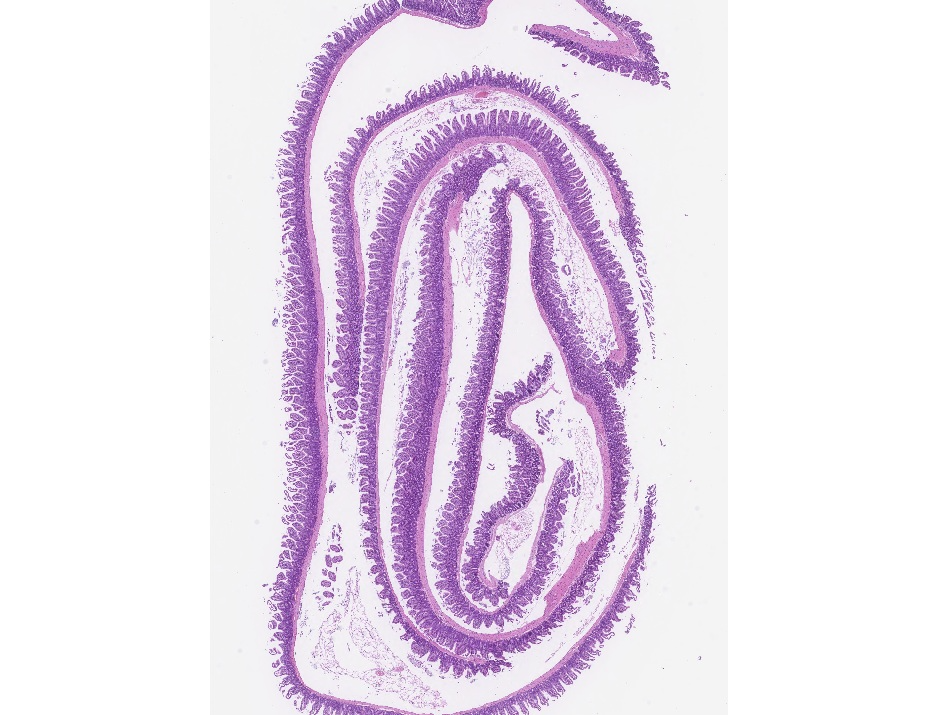

Supplement: Supplementary file 10 — Source data Fig. 1 [file 44319_2026_726_MOESM10_ESM.zip › Figure 1/Figure 1F-PKO 555-3 0.8x.jpg]

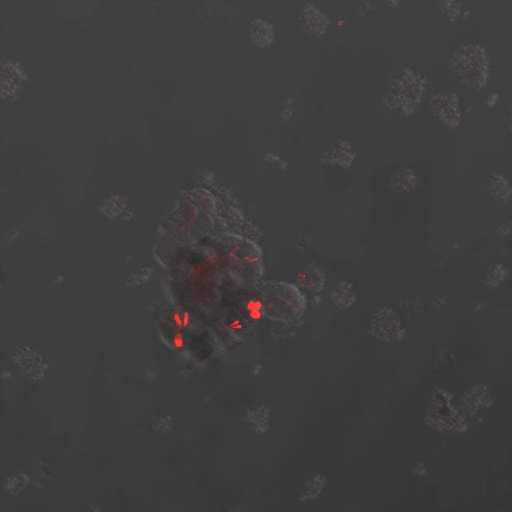

Supplement: Supplementary file 10 — Source data Fig. 1 [file 44319_2026_726_MOESM10_ESM.zip › Figure 1/Figure 1D-PHet 2.jpg]

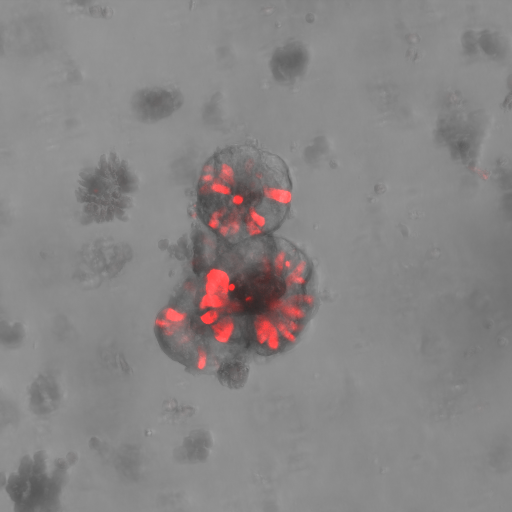

Supplement: Supplementary file 10 — Source data Fig. 1 [file 44319_2026_726_MOESM10_ESM.zip › Figure 1/Figure 1D-PKO 3.tif]

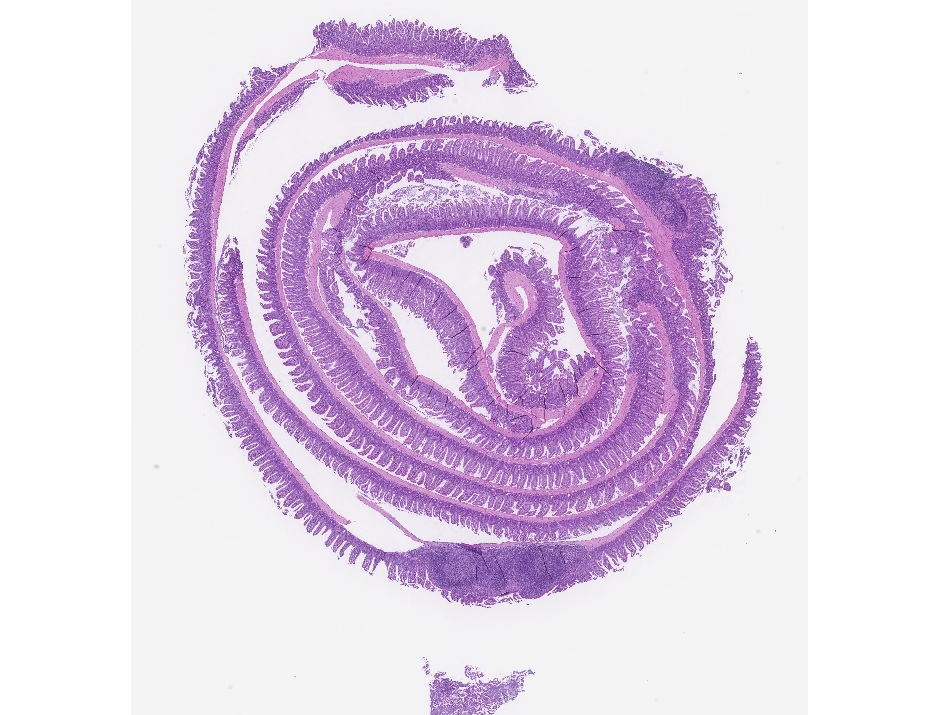

Supplement: Supplementary file 10 — Source data Fig. 1 [file 44319_2026_726_MOESM10_ESM.zip › Figure 1/Figure 1F-Flox 553-3 0.8x.jpg]

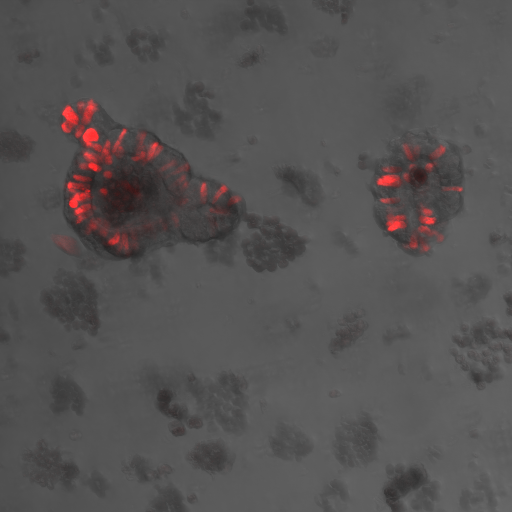

Supplement: Supplementary file 10 — Source data Fig. 1 [file 44319_2026_726_MOESM10_ESM.zip › Figure 1/Figure 1D-PKO 2.tif]

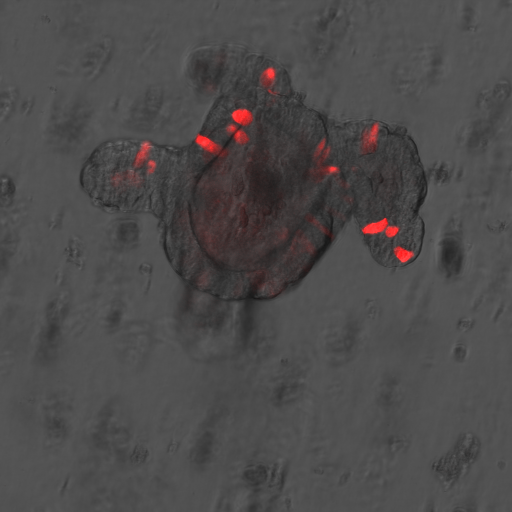

Supplement: Supplementary file 10 — Source data Fig. 1 [file 44319_2026_726_MOESM10_ESM.zip › Figure 1/Figure 1D-PKO 1.tif]

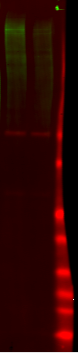

Supplement: Supplementary file 12 — Source data Fig. 3 [file 44319_2026_726_MOESM12_ESM.zip › Figure 3/Figure 3J-Ubiquitnation 2.tif]

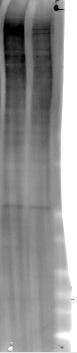

Supplement: Supplementary file 12 — Source data Fig. 3 [file 44319_2026_726_MOESM12_ESM.zip › Figure 3/Figure 3J-Ubiquitnation 1.tif]

**J**

### Acetylation and ubiquitination of $\beta$ -catenin in DLD1 cells (treated with TSA and MG-132)

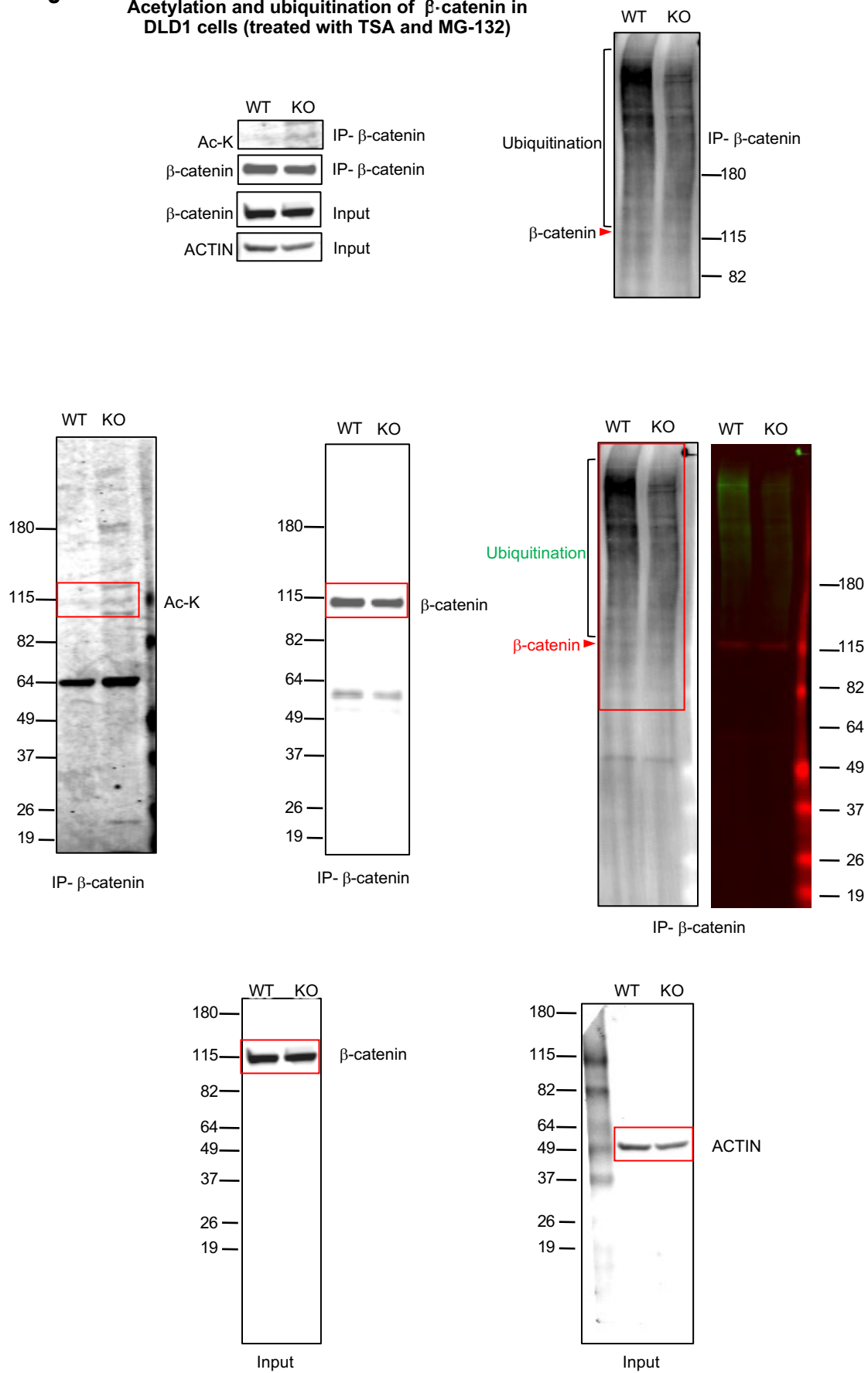

Supplement: Supplementary file 12 — Source data Fig. 3 [file 44319_2026_726_MOESM12_ESM.zip › Figure 3/Figure 3J-unprocessed immunoblots.pdf]

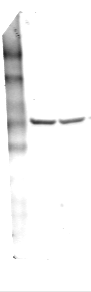

Supplement: Supplementary file 12 — Source data Fig. 3 [file 44319_2026_726_MOESM12_ESM.zip › Figure 3/Figure 3J-ACTIN.tif]

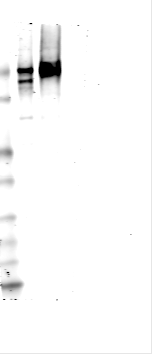

Supplement: Supplementary file 12 — Source data Fig. 3 [file 44319_2026_726_MOESM12_ESM.zip › Figure 3/Figure 3I-beta-catenin.tif]

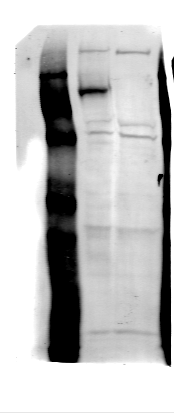

Supplement: Supplementary file 12 — Source data Fig. 3 [file 44319_2026_726_MOESM12_ESM.zip › Figure 3/Figure 3I-SIRT1.tif]

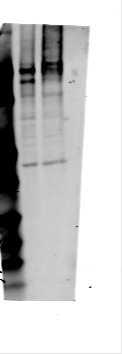

Supplement: Supplementary file 12 — Source data Fig. 3 [file 44319_2026_726_MOESM12_ESM.zip › Figure 3/Figure 3I-GAPDH.tif]

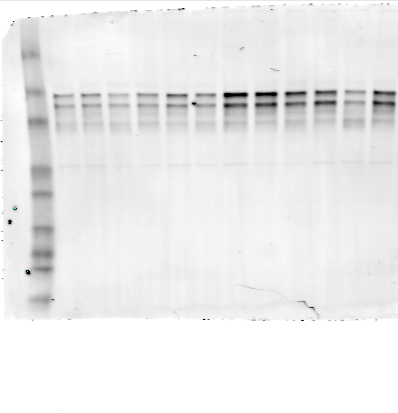

Supplement: Supplementary file 12 — Source data Fig. 3 [file 44319_2026_726_MOESM12_ESM.zip › Figure 3/Figure 3G-beta-catenin.tif]

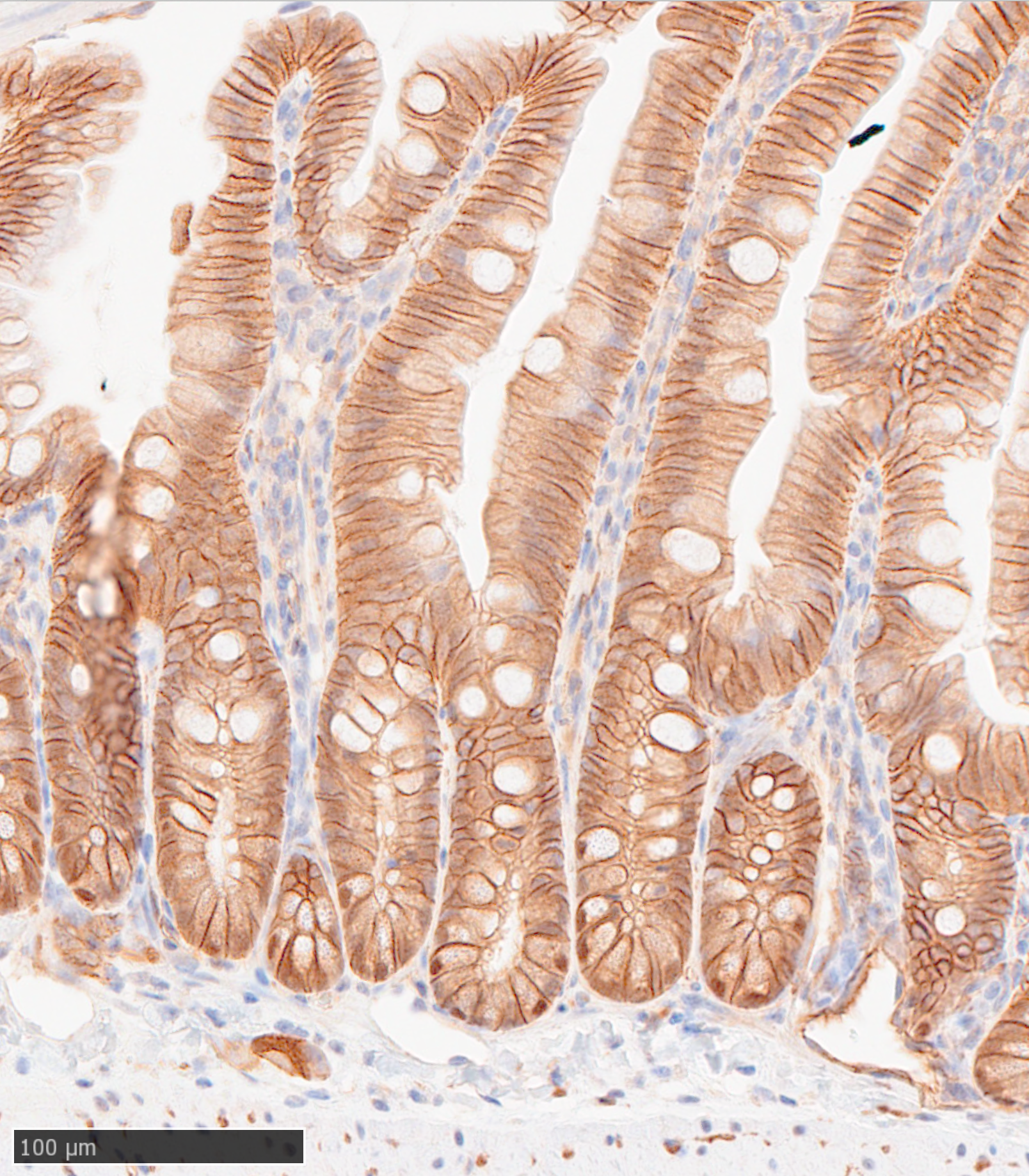

Supplement: Supplementary file 12 — Source data Fig. 3 [file 44319_2026_726_MOESM12_ESM.zip › Figure 3/Figure 3D-PKO 501-3 20x1.png]

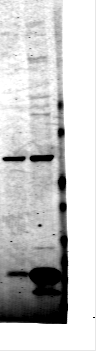

Supplement: Supplementary file 12 — Source data Fig. 3 [file 44319_2026_726_MOESM12_ESM.zip › Figure 3/Figure 3J-Ac-K.tif]

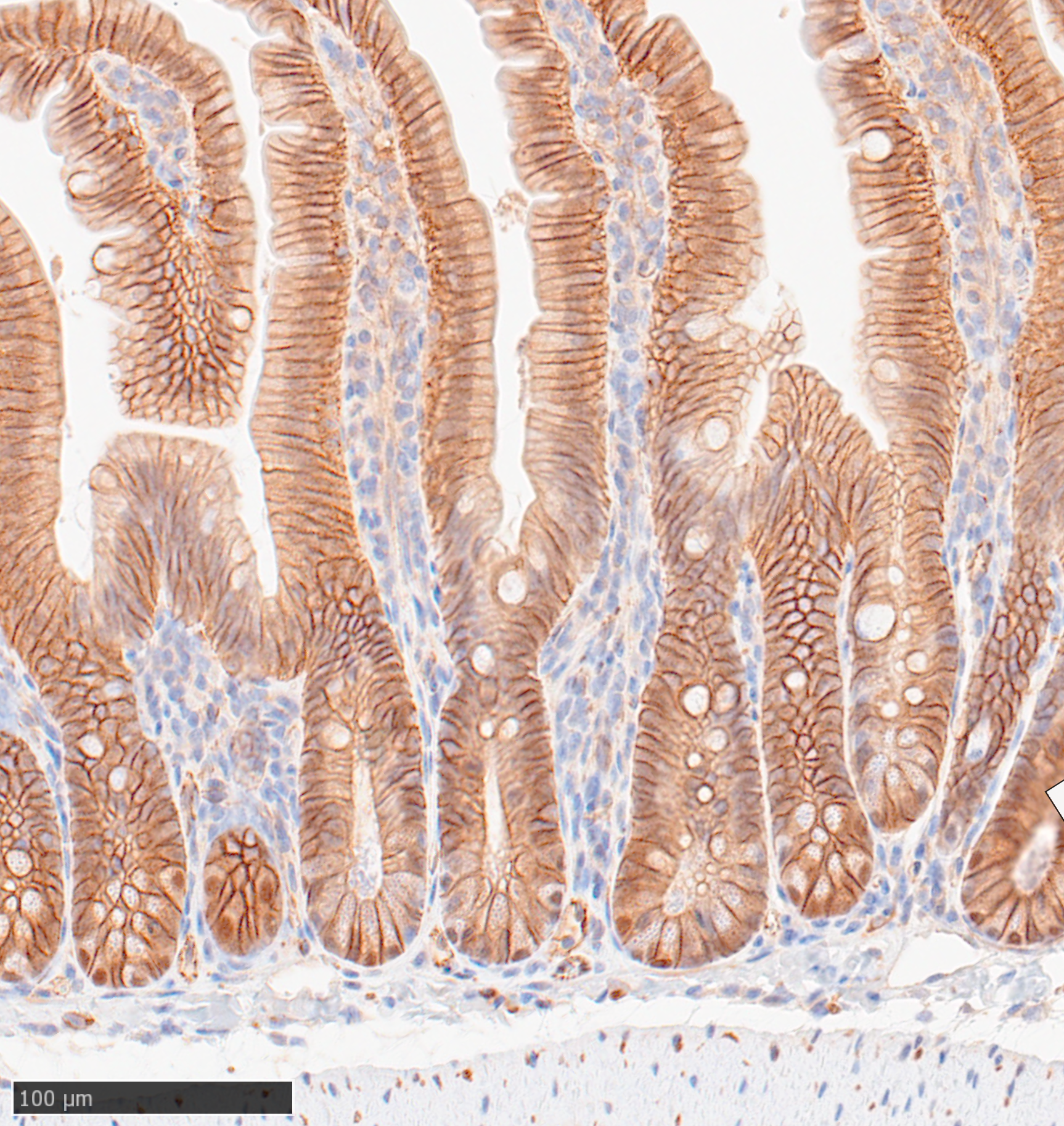

Supplement: Supplementary file 12 — Source data Fig. 3 [file 44319_2026_726_MOESM12_ESM.zip › Figure 3/Figure 3D-Flox 500-3 20x1.png]

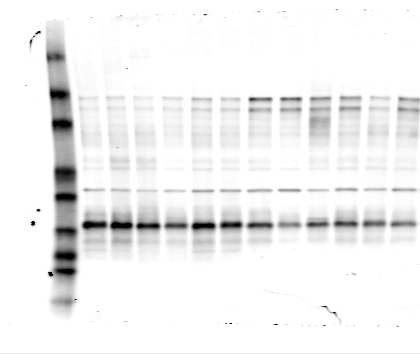

Supplement: Supplementary file 12 — Source data Fig. 3 [file 44319_2026_726_MOESM12_ESM.zip › Figure 3/Figure 3G-GAPDH.tif]

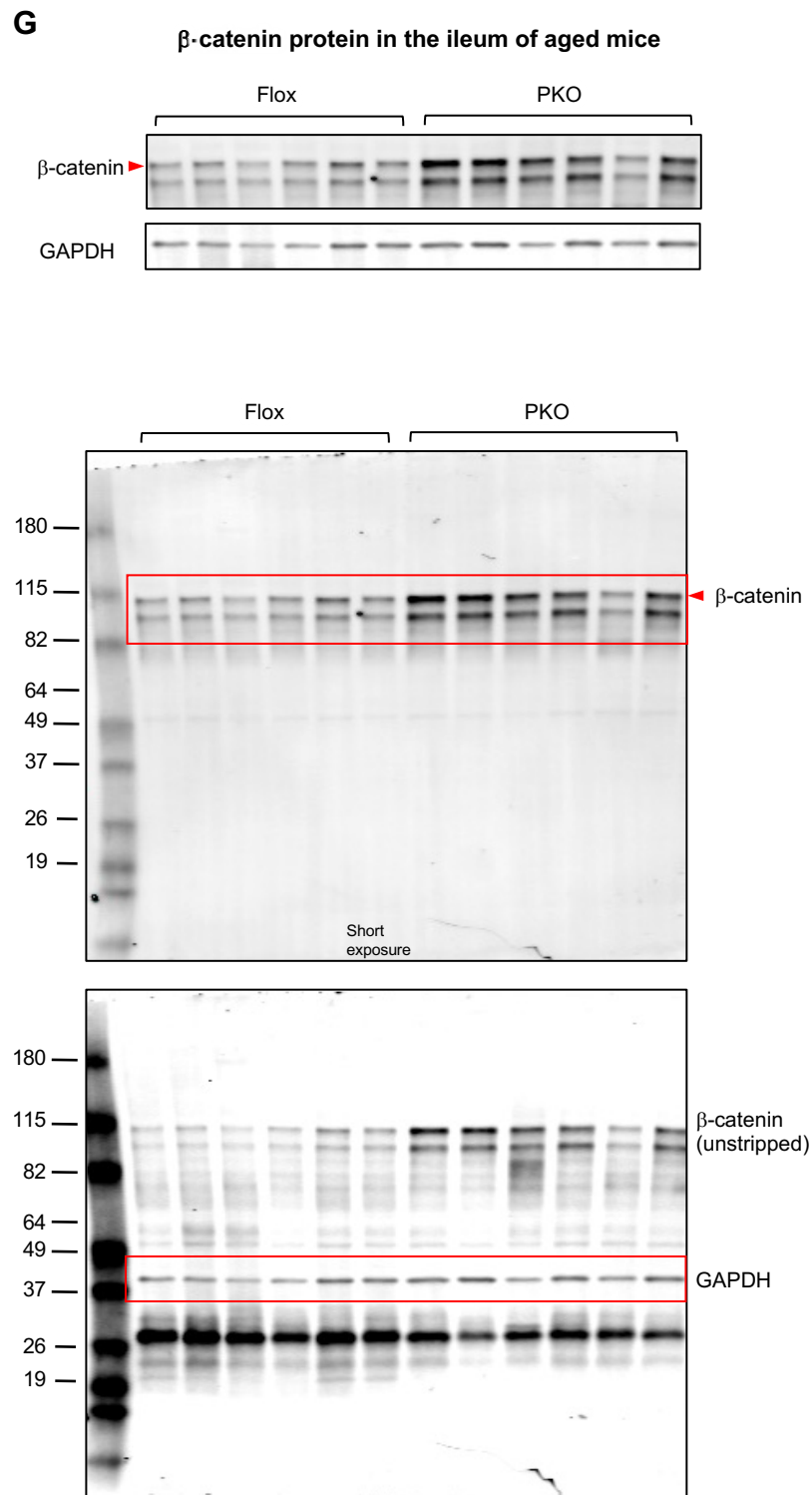

Supplement: Supplementary file 12 — Source data Fig. 3 [file 44319_2026_726_MOESM12_ESM.zip › Figure 3/Figure 3G-unprocessed immunoblots.pdf]

I  
**β-catenin protein  
in DLD1 cells**

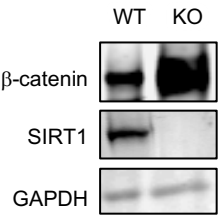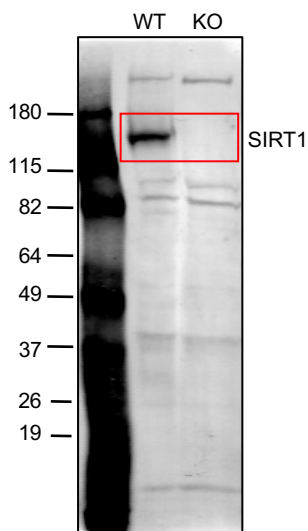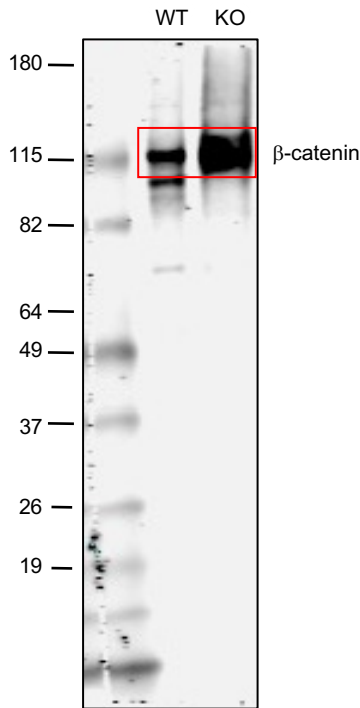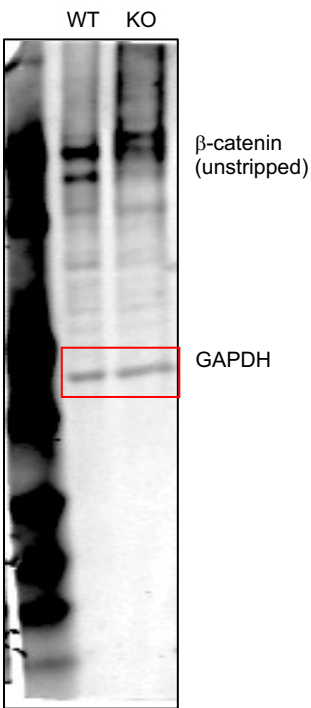

Supplement: Supplementary file 12 — Source data Fig. 3 [file 44319_2026_726_MOESM12_ESM.zip › Figure 3/Figure 3I-unprocessed immunoblots.pdf]

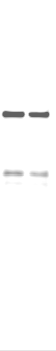

Supplement: Supplementary file 12 — Source data Fig. 3 [file 44319_2026_726_MOESM12_ESM.zip › Figure 3/Figure 3J-beta-catenin-IP.tif]

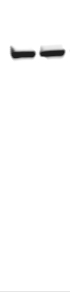

Supplement: Supplementary file 12 — Source data Fig. 3 [file 44319_2026_726_MOESM12_ESM.zip › Figure 3/Figure 3J-beta-catenin-input.tif]

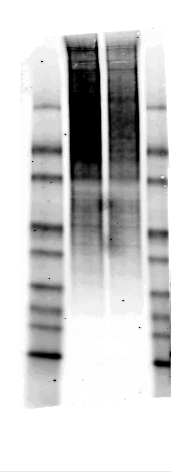

Supplement: Supplementary file 13 — Source data Fig. 4 [file 44319_2026_726_MOESM13_ESM.zip › Figure 4/Figure 4F-Ubiquitnation.tif]

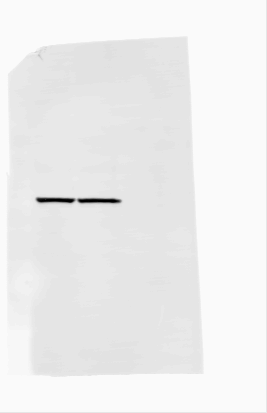

Supplement: Supplementary file 13 — Source data Fig. 4 [file 44319_2026_726_MOESM13_ESM.zip › Figure 4/Figure 4F-ACTIN.tif]

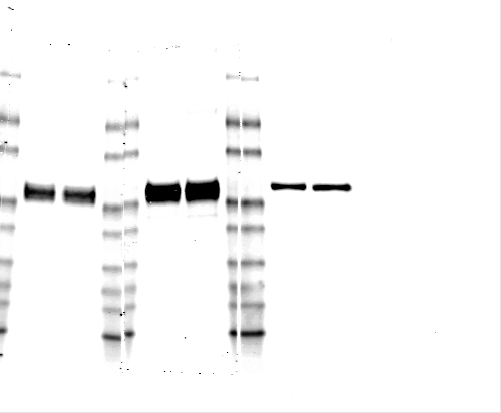

Supplement: Supplementary file 13 — Source data Fig. 4 [file 44319_2026_726_MOESM13_ESM.zip › Figure 4/Figure 4F-Ac-K, ATF4-IP, ATF4-Input.tif]

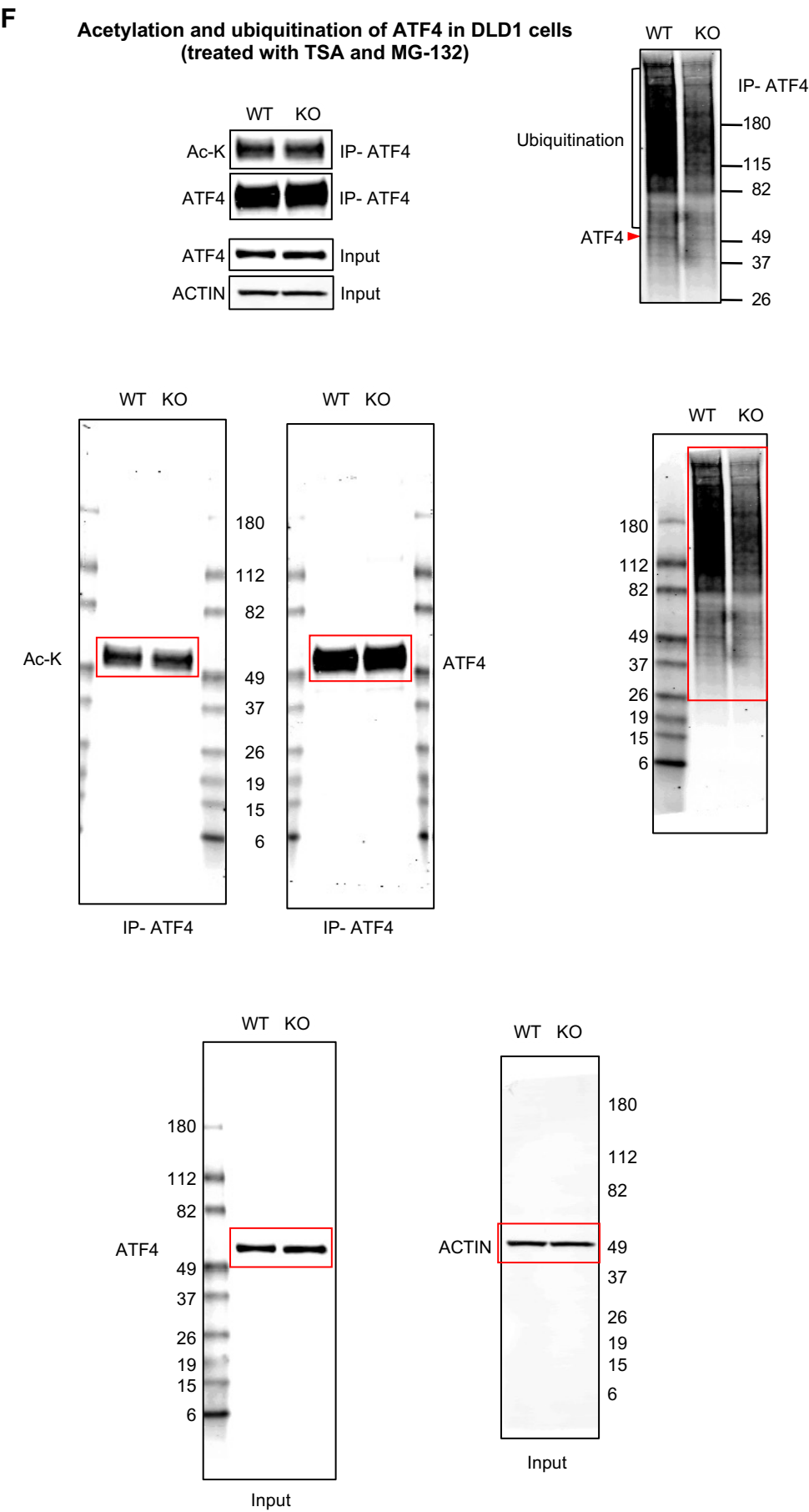

Supplement: Supplementary file 13 — Source data Fig. 4 [file 44319_2026_726_MOESM13_ESM.zip › Figure 4/Figure 4F-unprocessed immunoblots.pdf]

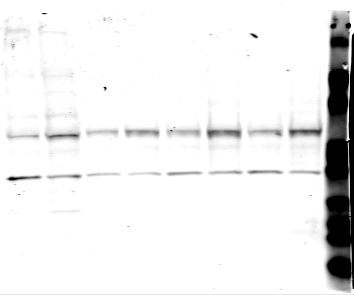

Supplement: Supplementary file 13 — Source data Fig. 4 [file 44319_2026_726_MOESM13_ESM.zip › Figure 4/Figure 4E-ATF4 ACTIN.tif]

**E**      **ATF4 protein levels upon stress in DLD1 cells**

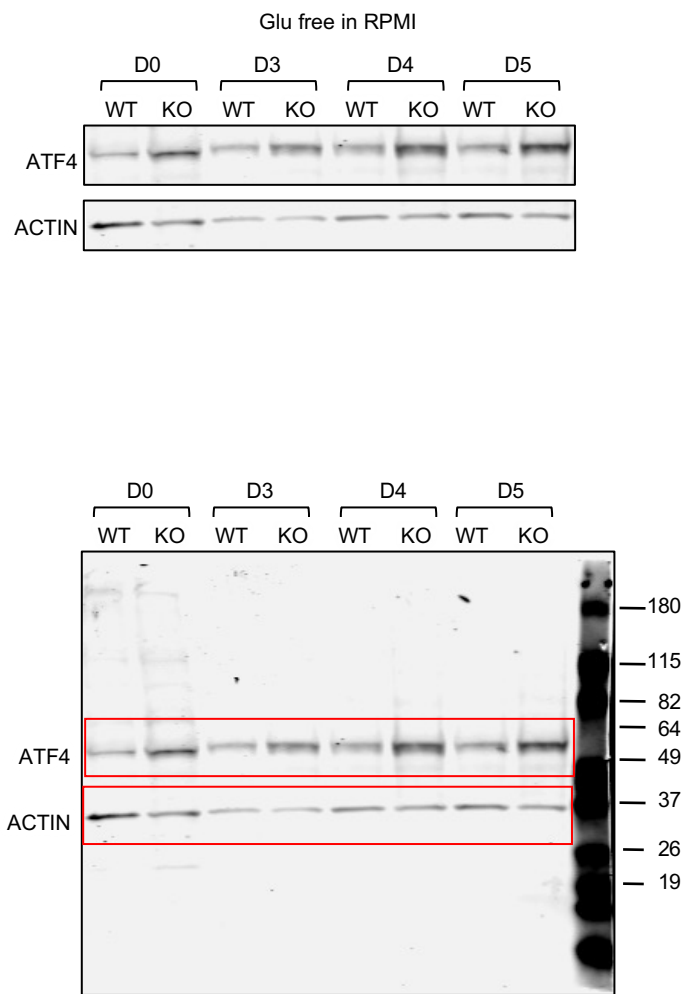

Supplement: Supplementary file 13 — Source data Fig. 4 [file 44319_2026_726_MOESM13_ESM.zip › Figure 4/Figure 4E-unprocessed immunoblots.pdf]

#### 4D Cell death in DLD1 cells

Glu free in RPMI

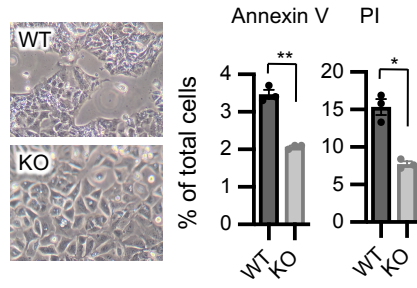

WT

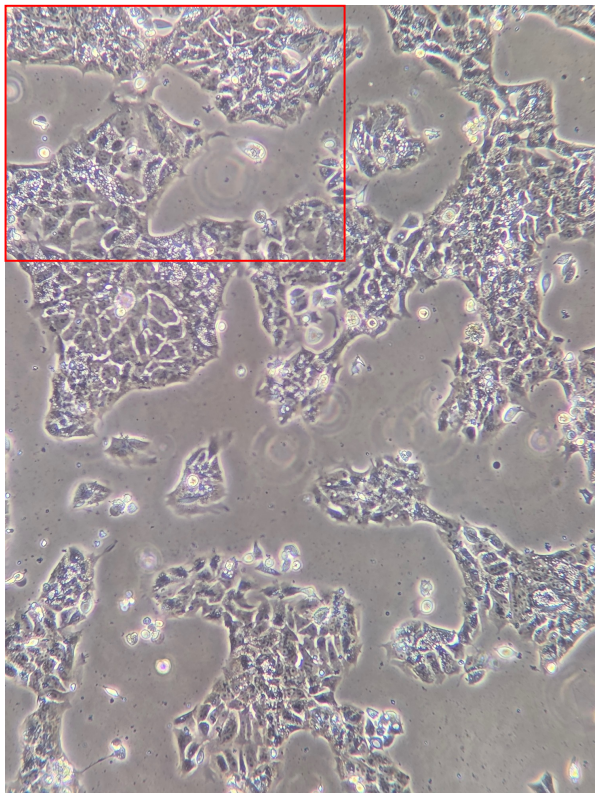

KO

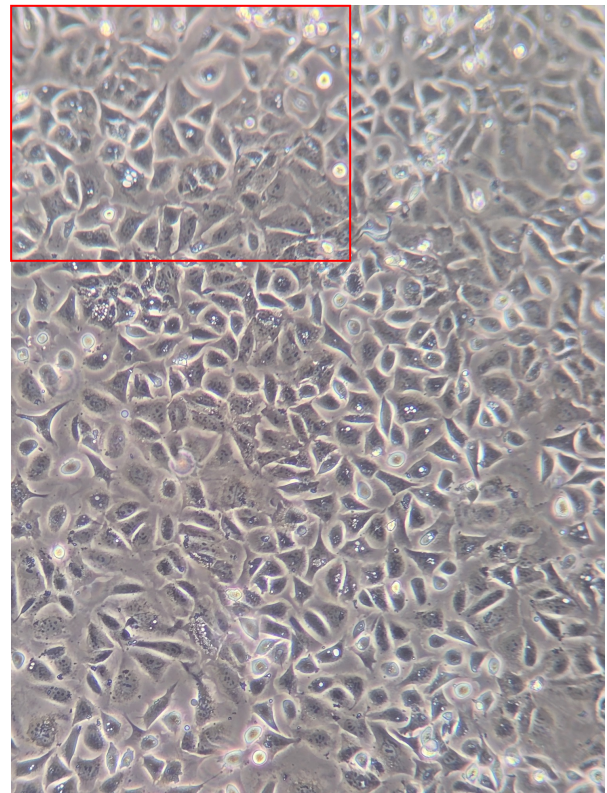

Supplement: Supplementary file 13 — Source data Fig. 4 [file 44319_2026_726_MOESM13_ESM.zip › Figure 4/Figure 4D-uncut images.pdf]

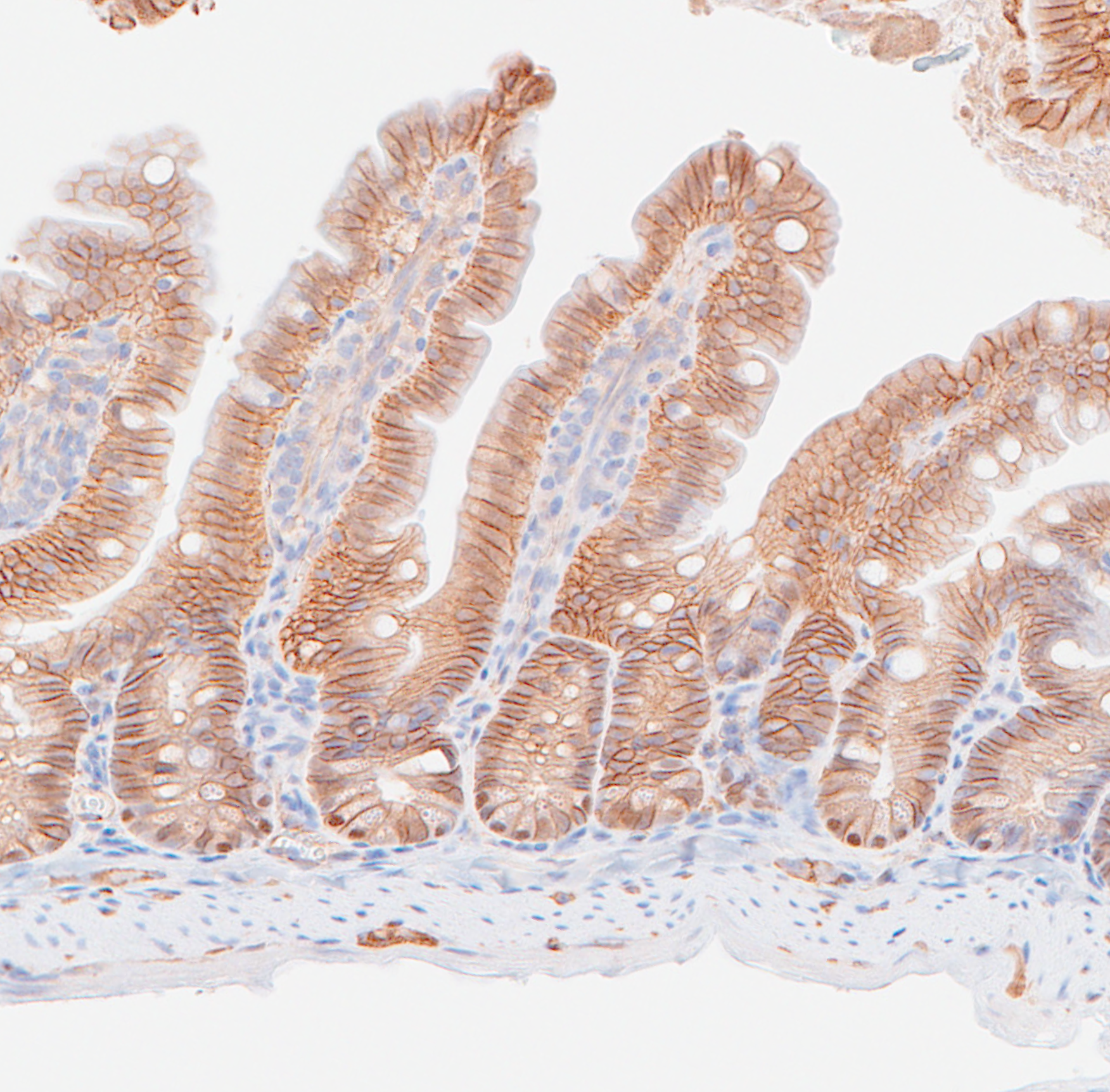

Supplement: Supplementary file 15 — Source data Fig. 6 [file 44319_2026_726_MOESM15_ESM.zip › Figure 6/Figure 6H-PKO 439-3-20x2.png]

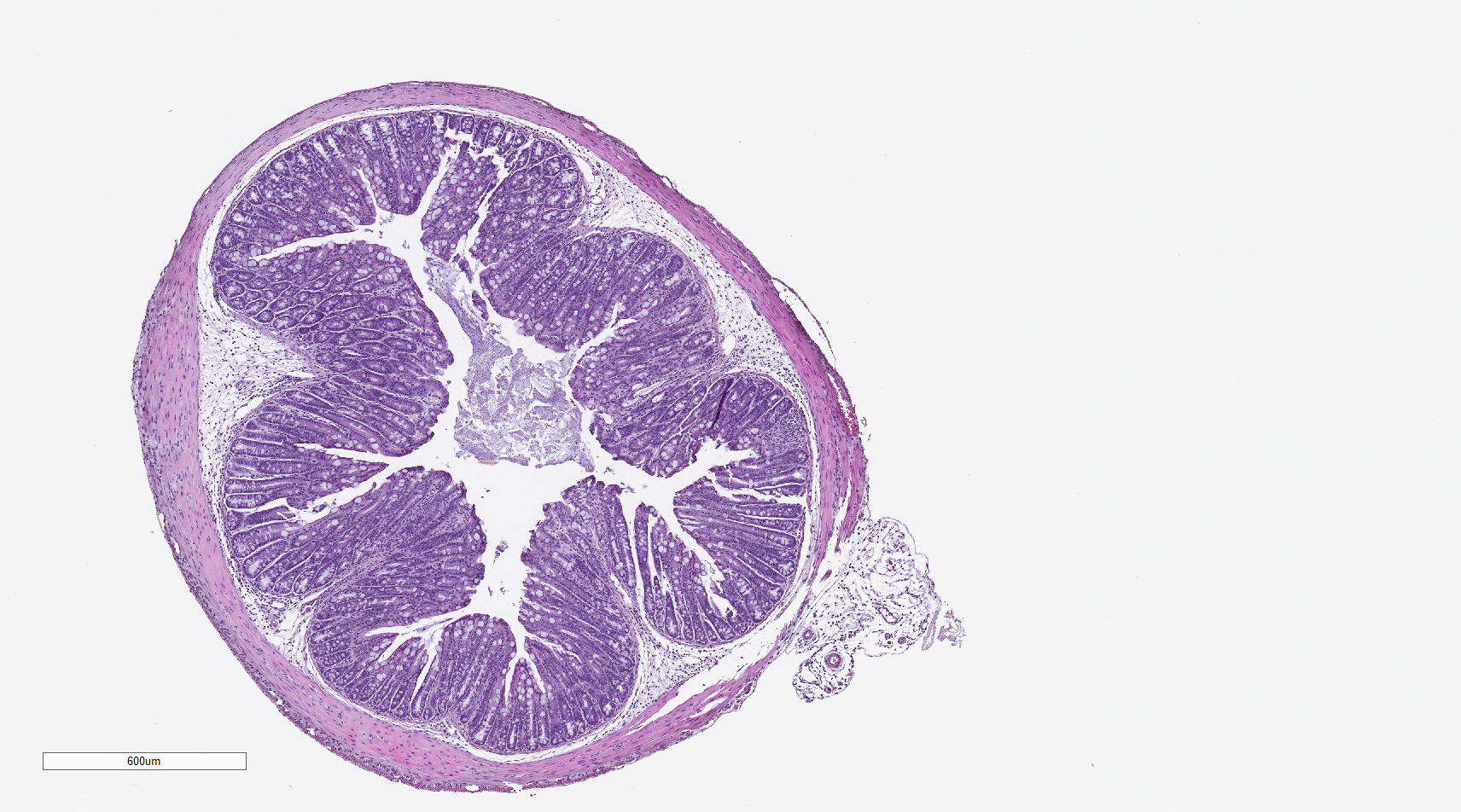

Supplement: Supplementary file 15 — Source data Fig. 6 [file 44319_2026_726_MOESM15_ESM.zip › Figure 6/Figure 6D PKO-H&E-428_4X_SB.tif]

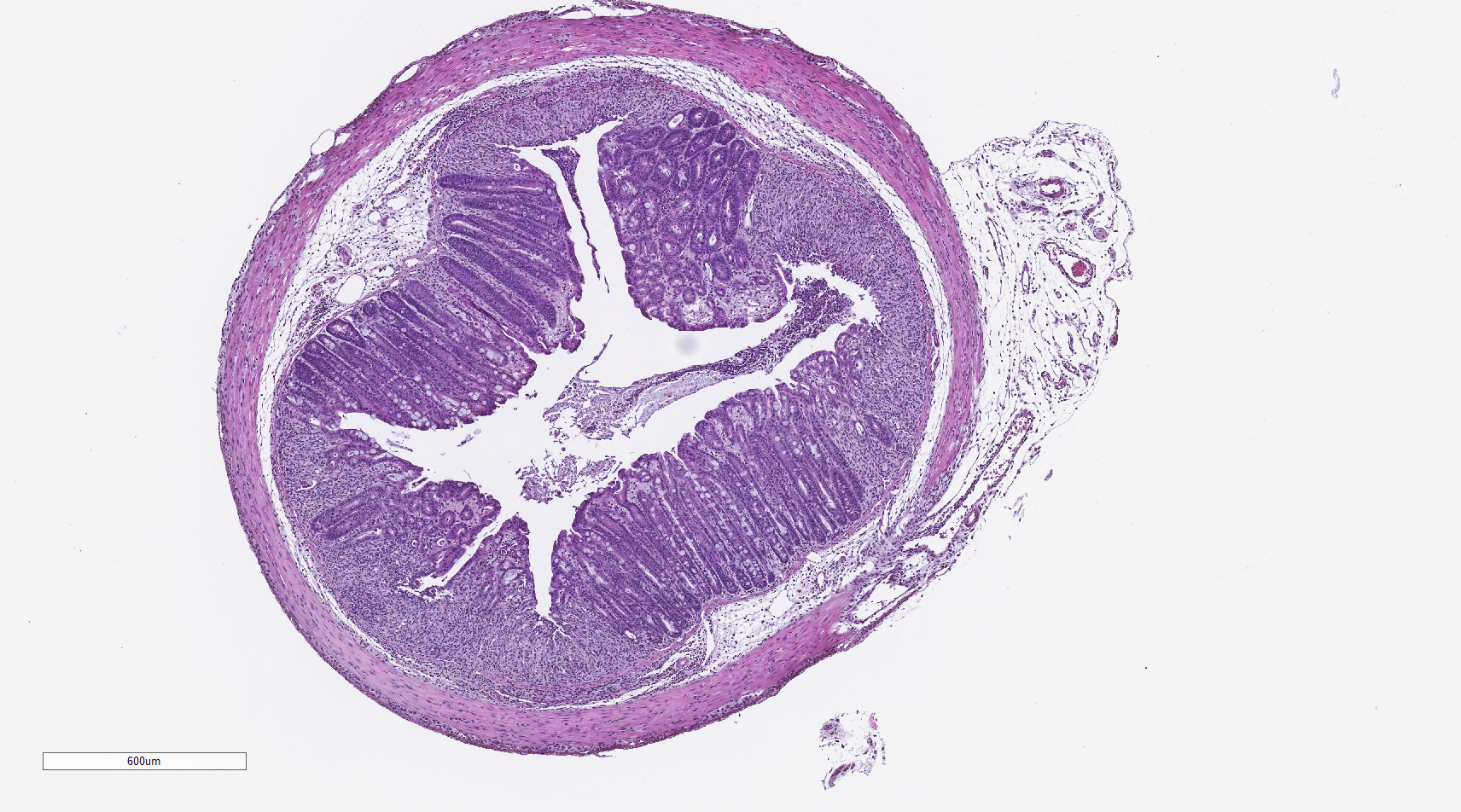

Supplement: Supplementary file 15 — Source data Fig. 6 [file 44319_2026_726_MOESM15_ESM.zip › Figure 6/Figure 6D Flox-H&E-410_4x_SB.tif]

**H**  $\beta$ -catenin in the ileum  
(9 month old)

Flox

PKO

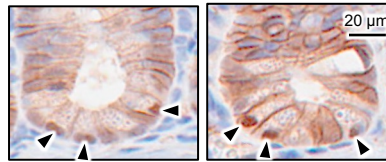

Flox

PKO

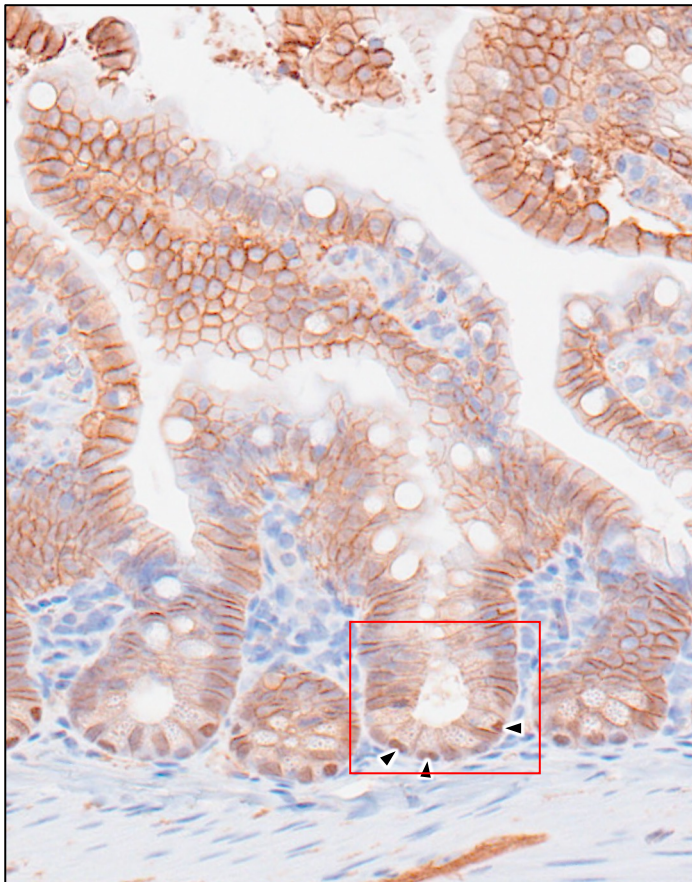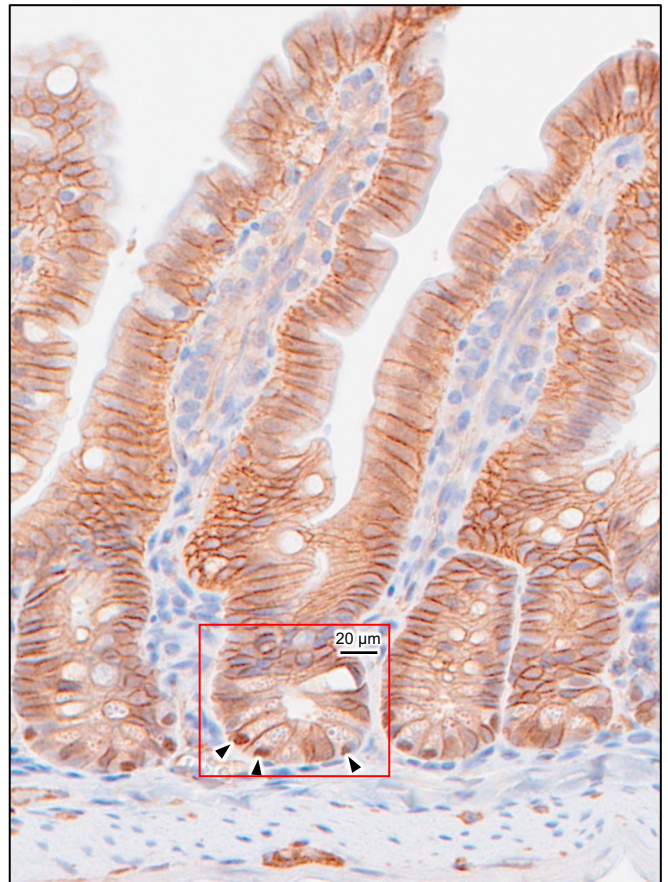

Supplement: Supplementary file 15 — Source data Fig. 6 [file 44319_2026_726_MOESM15_ESM.zip › Figure 6/Figure 6H.pdf]

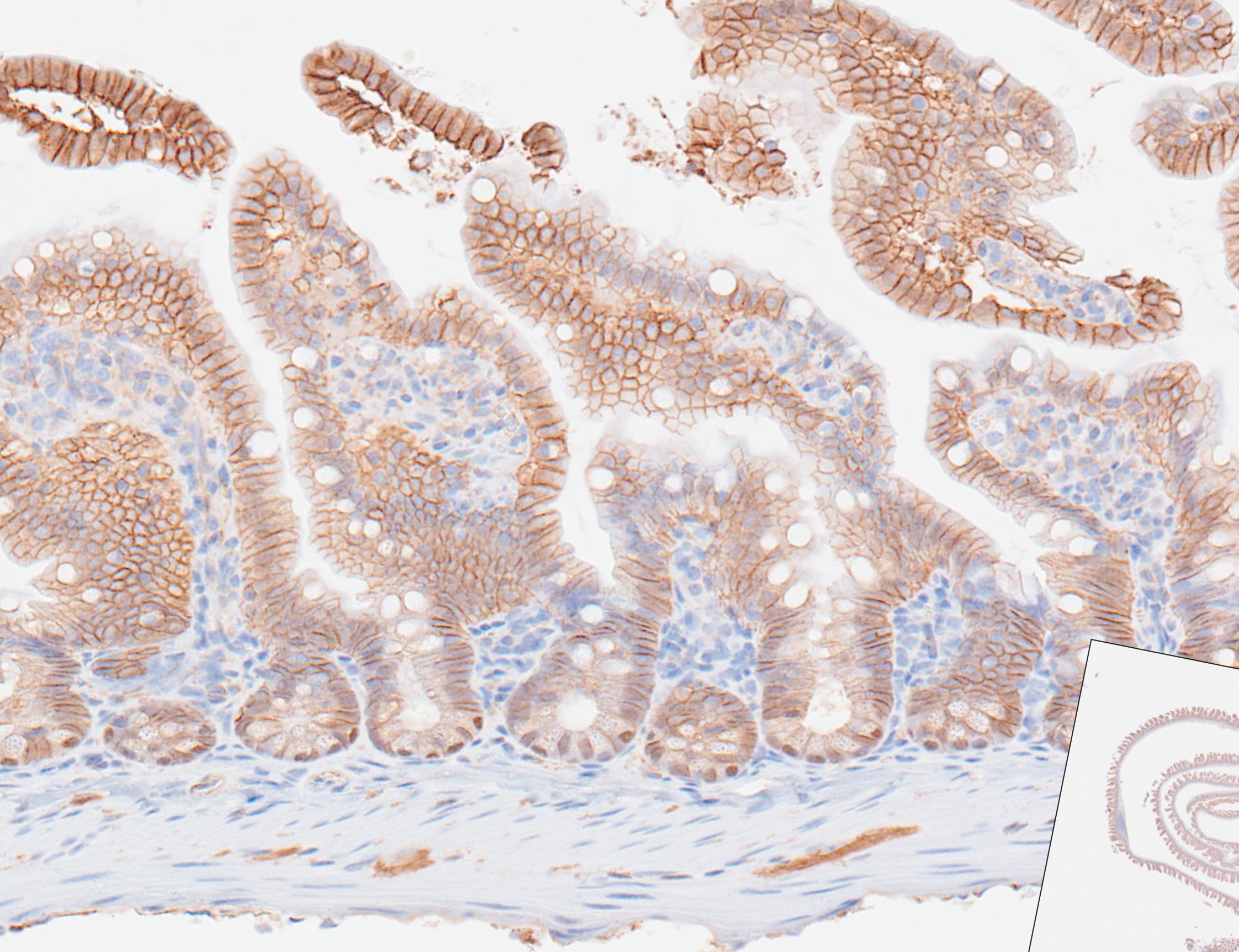

Supplement: Supplementary file 15 — Source data Fig. 6 [file 44319_2026_726_MOESM15_ESM.zip › Figure 6/Figure 6H-Flox 440-3-20x1.png]

71 Colonic tissue histology

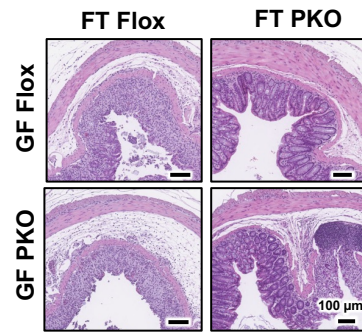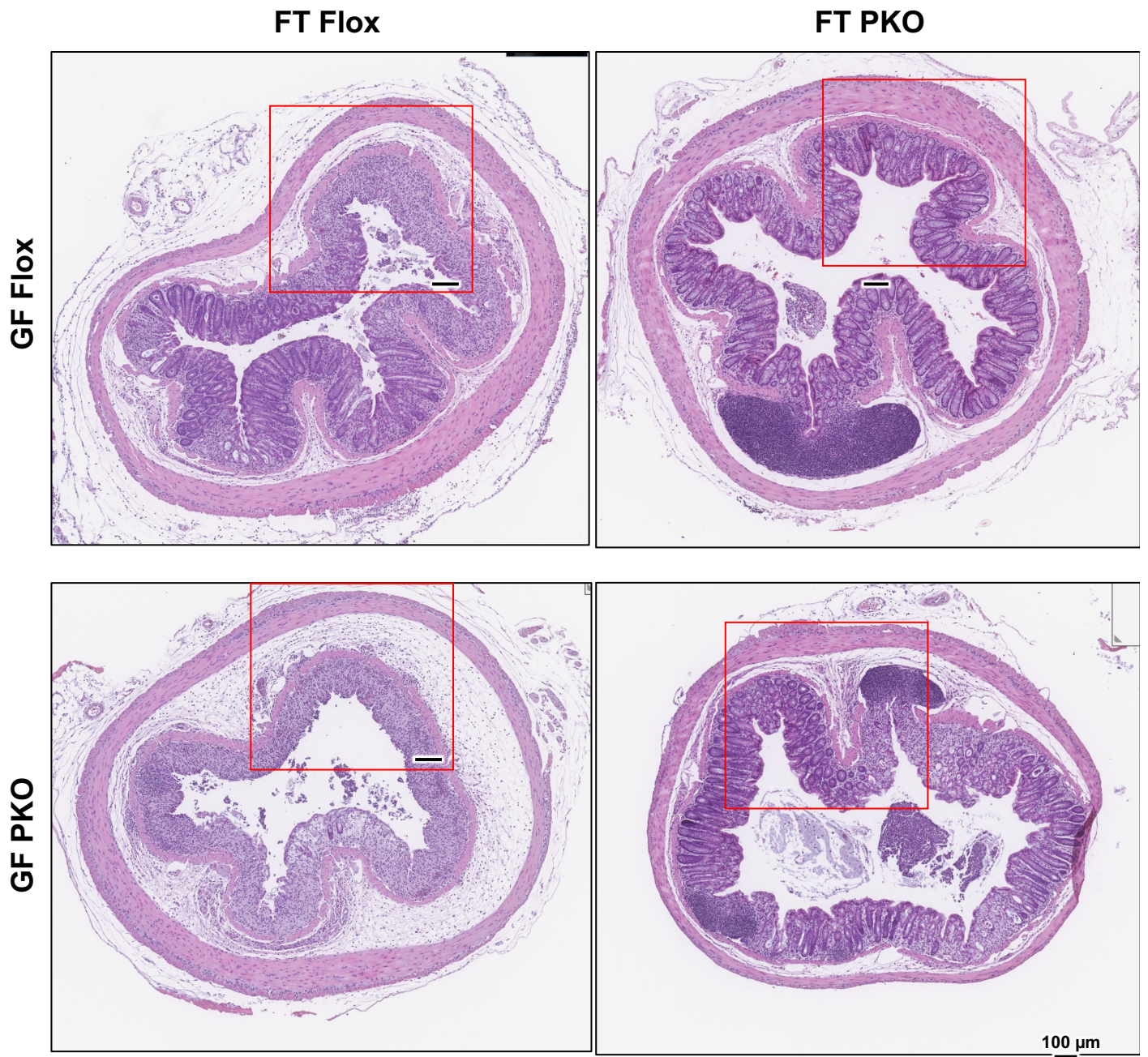

Supplement: Supplementary file 16 — Source data Fig. 7 [file 44319_2026_726_MOESM16_ESM.zip › Figure 7/Figure 7I.pdf]

7D

Colonic tissue histology

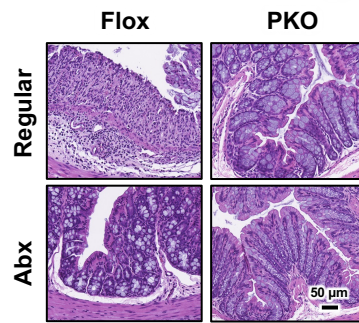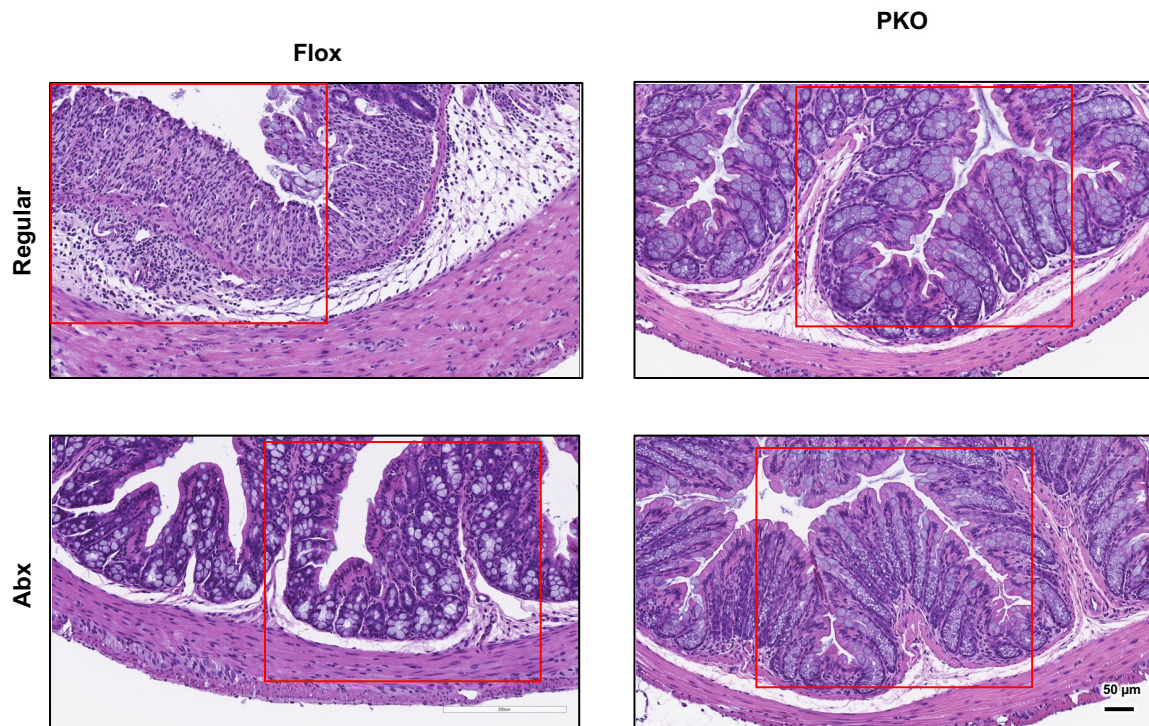

Supplement: Supplementary file 16 — Source data Fig. 7 [file 44319_2026_726_MOESM16_ESM.zip › Figure 7/Figure 7D.pdf]
