# Supplementary material for: Paneth cell SIRT1 deficiency increases intestinal stress resistance by modulating the gut microbiota
Source: EMBO Rep. 2026 Mar 13;27(7):1830–57. doi: 10.1038/s44319-026-00726-3 (PMC13076647; doi:10.1038/s44319-026-00726-3)
Supplement: Supplementary file 23 — Expanded View Figures [file 44319_2026_726_MOESM23_ESM.pdf]

Expanded View Figures

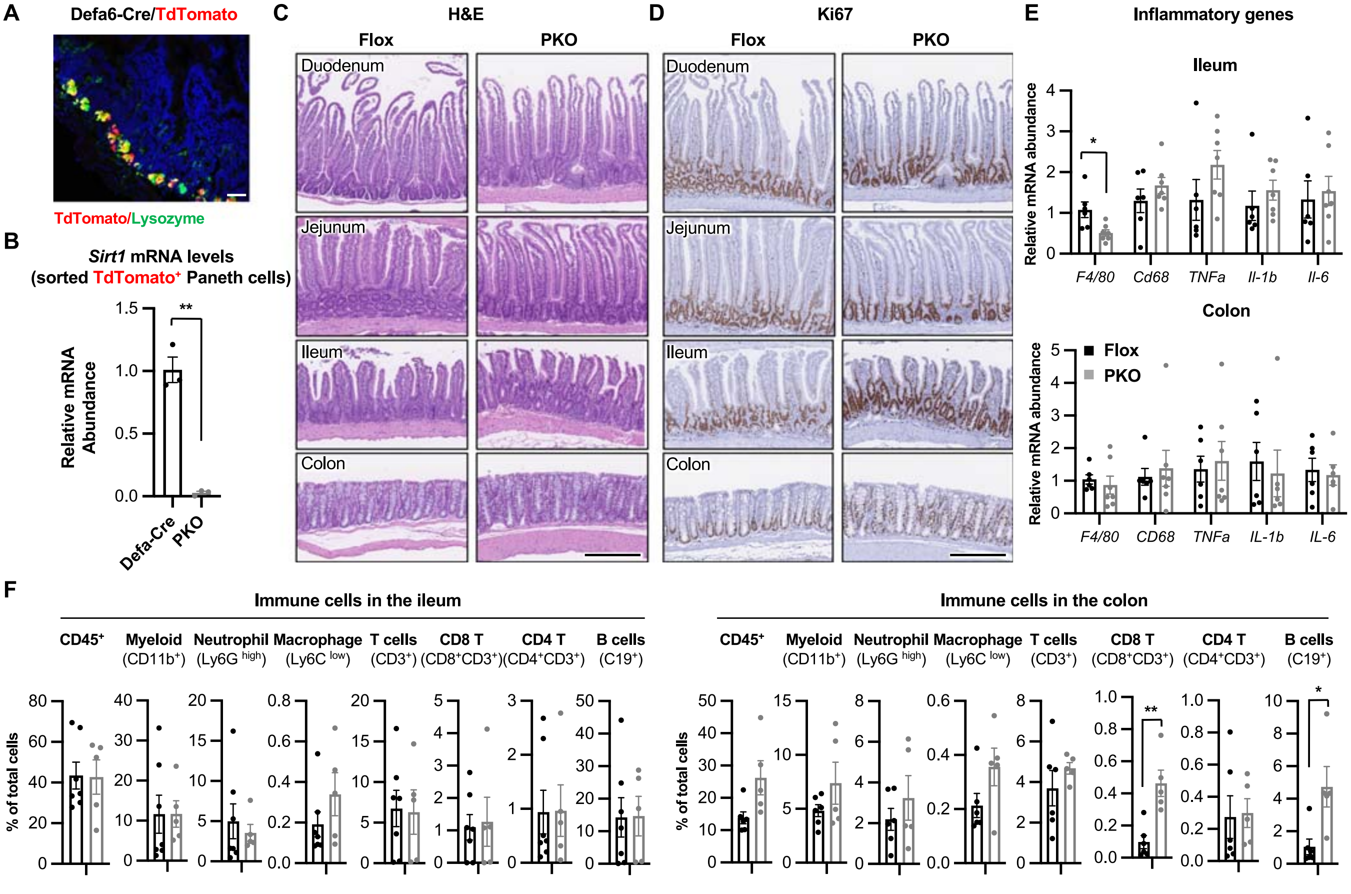

Figure EV1. Young SIRT1 PKO mice have normal gut biology under standard feeding conditions.

(**A**) Defa6Cre drives specific expression of TdTomato in Paneth cells. The expression of TdTomato and lysozyme in the ileum of Defa6-Cre/TdTomato mice were analyzed by IF. Bar, 50 µm. (**B**) *Sirt1* is efficiently deleted in Paneth cells in SIRT1 PKO mice. Paneth cells in Defa6-Cre control and PKO mice on a TdTomato+ background were sorted out and the levels of *Sirt1* mRNA in sorted Paneth cells were analyzed by qPCR ($n = 3$ pairs of mice, paired t-test). (**C**) H&E staining of intestinal sections of young Flox and SIRT1 PKO mice. Bars, 250 µm. (**D**) Young Flox and SIRT1 PKO mice have comparable cell proliferation under normal feeding condition. Intestinal sections from 4-month-old Flox and SIRT1 PKO mice were stained with an anti-Ki67 antibody. (**E**) Young Flox and SIRT1 PKO mice have comparable expression of inflammatory genes in the gut under normal feeding condition. The expression of indicated genes was analyzed by qPCR ($n = 6$ Flox and 7 PKO, Student's t-test). (**F**) Immune cells from the ileum and colon of young Flox and SIRT1 PKO mice. The fraction of indicated immune cells in total live cells isolated from Flox and PKO mice was analyzed by FACS as described in Methods (Ileum: $n = 7$ Flox and 5 PKO mice; Colon: $n = 6$ Flox and 5 PKO mice, Student's t-test). Data information: in (**B**, **E**, and **F**), values are expressed as mean ± SEM; $*p < 0.05$, $**p < 0.01$; no marks, not significant. Source data are available online for this figure.

This is a U.S. Government work and not under copyright protection in the US; foreign copyright protection may apply

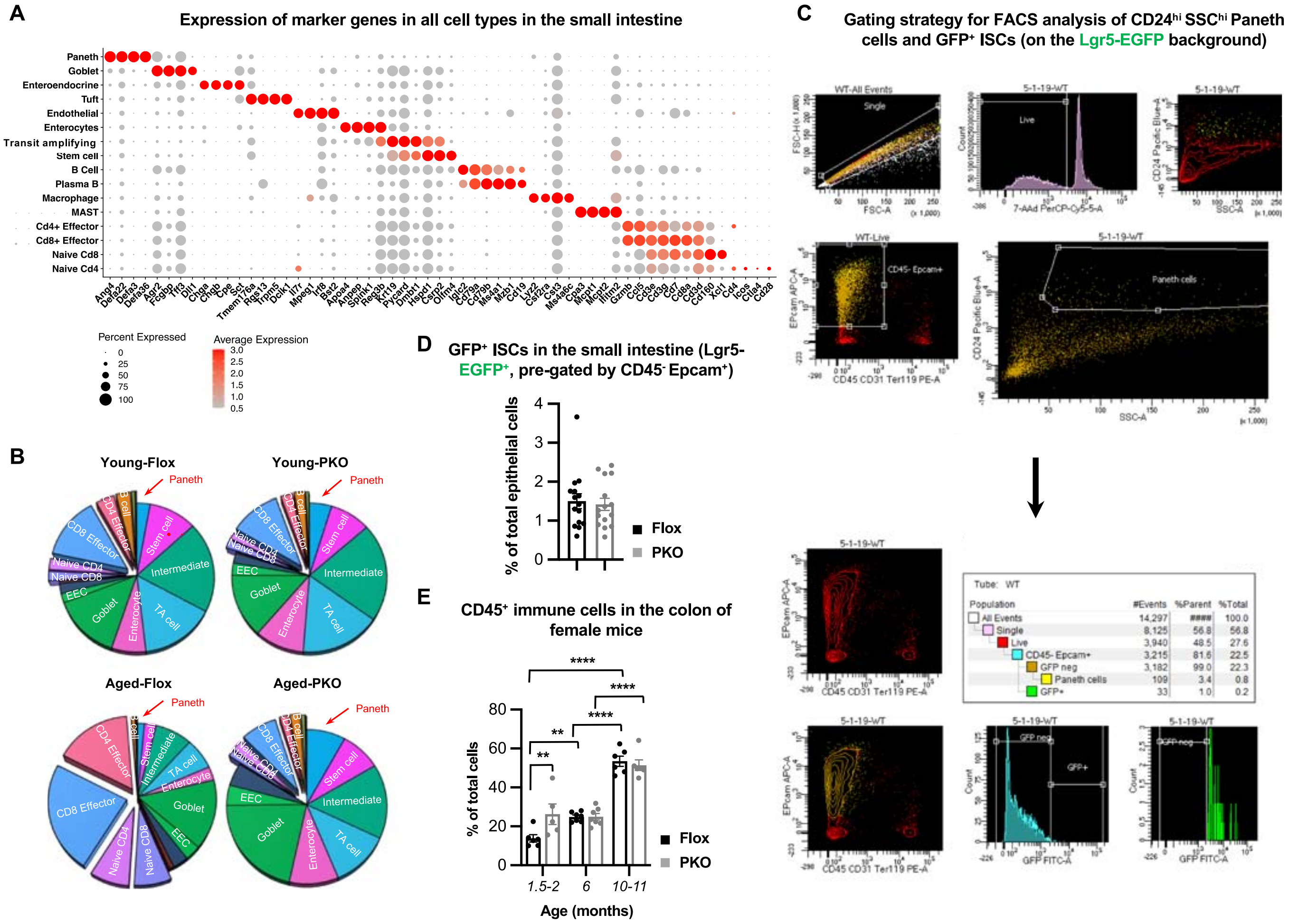

Figure EV2. scRNA-seq analysis of small intestinal cells in young and aged Flox and SIRT1 PKO mice.

(**A**) Dot plot showing the expression of marker genes in different function groups of total live cells isolated from the small intestine. (**B**) Relative composition of different cell types in the small intestine of young and aged mice. (**C**) Gating strategy for FACS analysis of CD24^{hi} SSC^{hi} Paneth cells and GFP^{+} intestinal stem cells (ISCs). Small intestinal epithelial cells from 2- to 3-month-old Flox and SIRT1 PKO mice on the Lgr5-EGFP background were isolated and analyzed as described in Methods. The FACS plots from the same Flox mouse in Fig. 1C are used here to demonstrate the gating strategy. (**D**) SIRT1 PKO mice have normal abundance of ISCs in the small intestine. The abundance of ISCs in Flox and PKO mice on the Lgr5-GFP background was analyzed by FACS ($n = 15$ pairs, paired t-test). (**E**) Age induces immune cell expansion in the colon. The fraction of CD45^{+} immune cells in total live cells isolated from Flox and PKO mice at indicated ages was analyzed by FACS as described in Methods ($n = 6, 5, 7, 6, 6, 6$ mice per indicated group, two-way ANOVA). Data information: (**D** and **E**), values are expressed as mean ± SEM; *$p < 0.05$, **$p < 0.01$, ****$p < 0.0001$; no marks, not significant. Source data are available online for this figure.

This is a U.S. Government work and not under copyright protection in the US; foreign copyright protection may apply

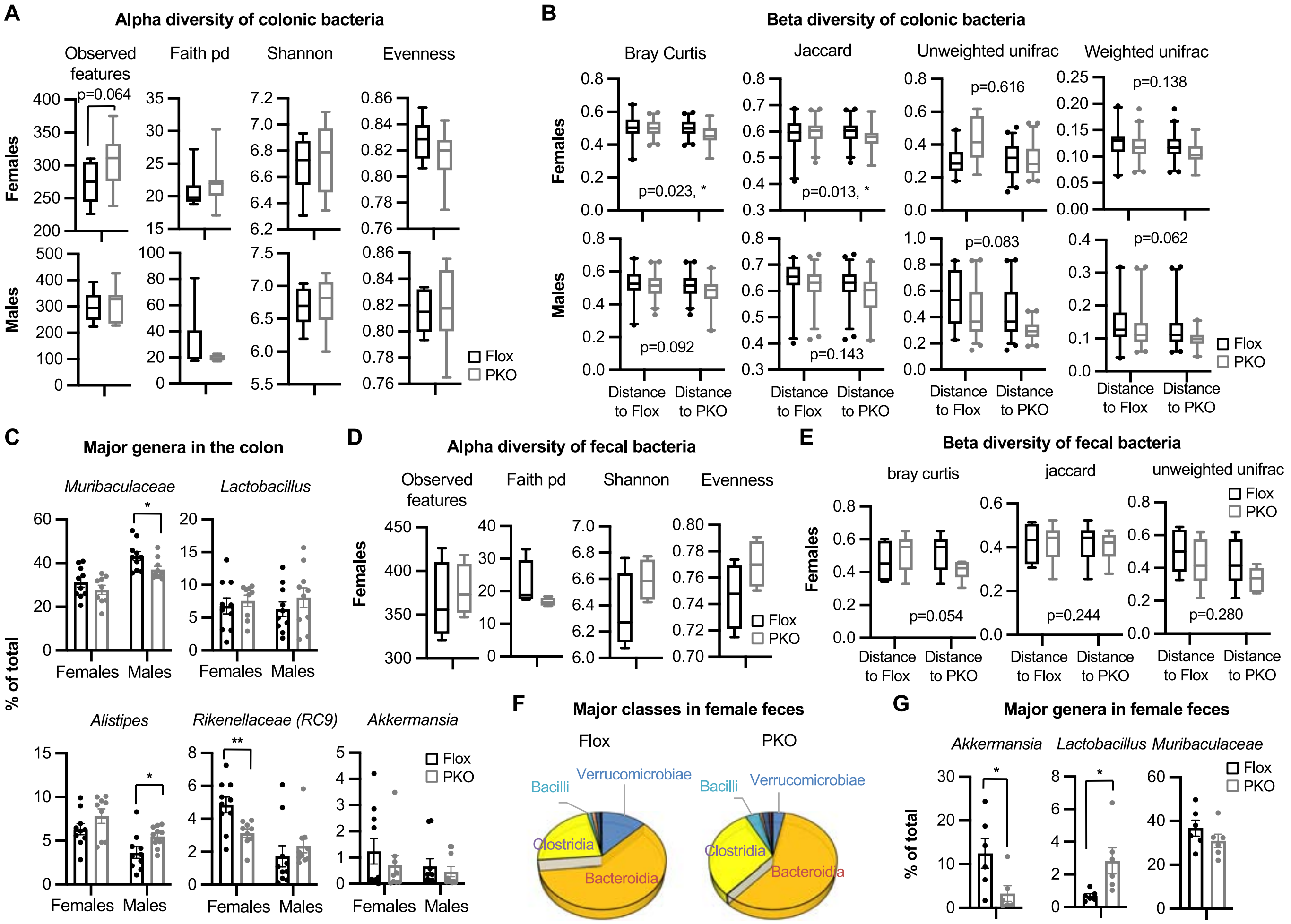

Figure EV3. The impact of Paneth cell SIRT1 deficiency on colonic and fecal bacteria.

(**A–C**) SIRT1 PKO mice have altered colonic microbiota. Total colonic DNA from Flox and SIRT1 PKO mice were analyzed for mucosal adherent microbiota using 16S rRNA gene amplicon sequencing as described in Methods ($n = 10$ Flox and 9 PKO females and 10 Flox and 10 PKO males). (**A**) Alpha diversity of colonic bacteria (Student's t-test). (**B**) Beta diversity of colonic bacteria (Pair-wise permanova test). (**C**) The abundance of major genera in the colon ($n = 10$ Flox and 9 PKO females and 10 Flox and 10 PKO males, Student's t-test). (**D–G**) SIRT1 PKO female mice have altered fecal microbiota. Total fecal DNA from Flox and SIRT1 PKO females were analyzed using 16S rRNA gene amplicon sequencing. (**D**) Alpha diversity of fecal bacteria ($n = 6$ mice/genotype, Student's t-test). (**E**) Beta diversity of fecal bacteria ($n = 6$ mice/genotype, Pair-wise permanova test). (**F**) Altered abundance of several major classes of fecal microbiota in SIRT1 PKO females. (**G**) SIRT1 PKO females have altered levels of several major genera in feces ($n = 6$/genotype, Student's t-test). Data information: in (**A**, **B**, **D**, and **E**), box-and-whisker plot with the box representing the interquartile range (Q1 to Q3), a line inside indicating the median, and whiskers representing the 2.5–97.5 percentile; in (**C** and **G**), values are expressed as mean ± SEM; *$p < 0.05$, **$p < 0.01$; no marks, not significant. Source data are available online for this figure.

This is a U.S. Government work and not under copyright protection in the US; foreign copyright protection may apply

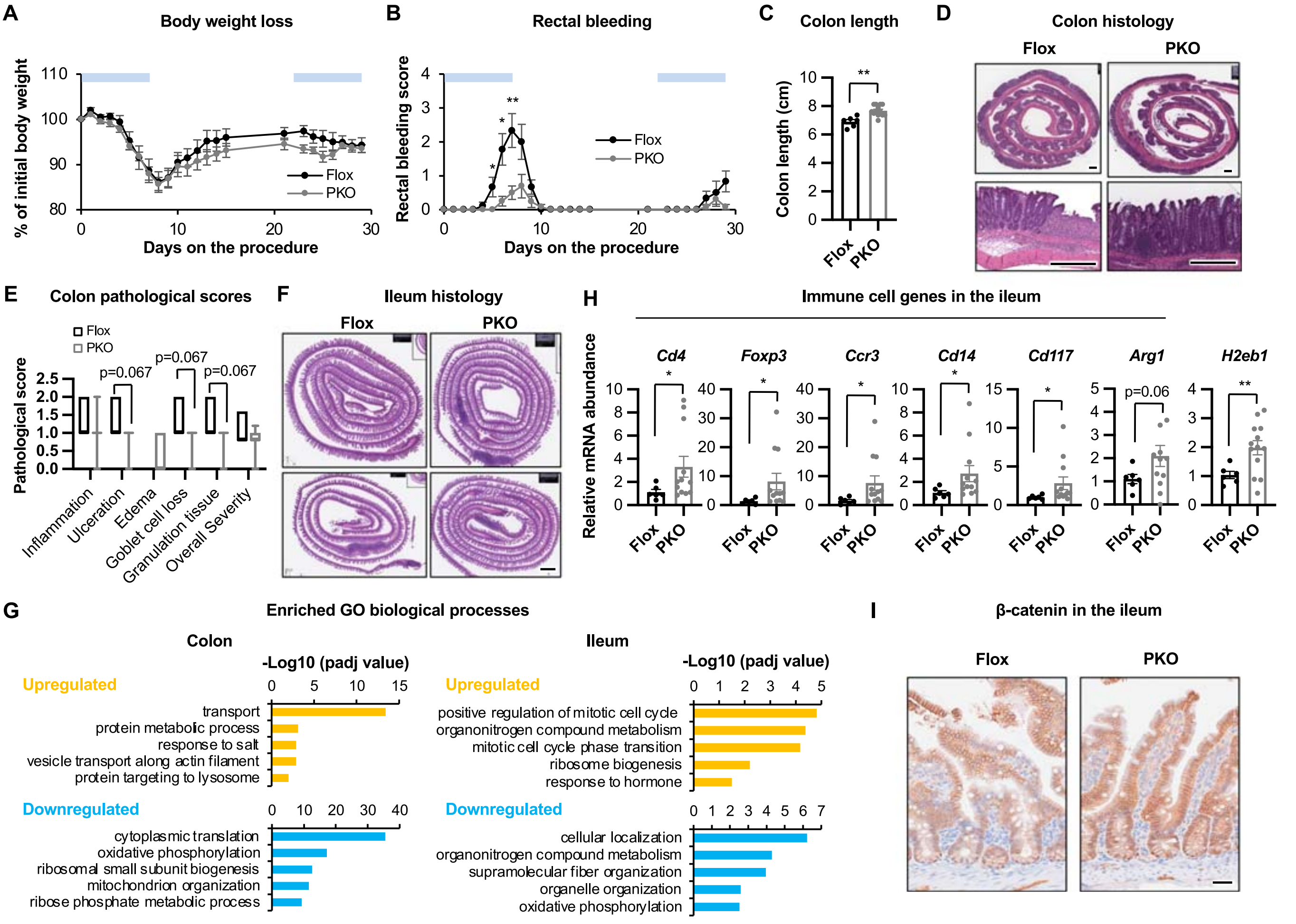

Figure EV4. SIRT1 PKO mice are protected from DSS-colitis.

(**A–C**) Nine-month-old SIRT1 PKO mice are more resistant to DSS-induced colitis than Flox mice. Nine-month-old Flox and SIRT1 PKO mice were treated with 2 cycles of 2.5% DSS for 7 days with 14 day of regular water break in between. Their body weight (**A**), rectal bleeding (**B**), and colon length (**C**) were analyzed ($n = 6$ Flox and 12 PKO mice, Student's t-test). (**D, E**) Colon histology of 9-month-old Flox and SIRT1 PKO mice after 2 cycles of DSS treatment. (**D**) Representative H&E staining of Swiss swirl from 2 Flox and 2 PKO mice are shown. Bars, 500 µm. (**E**) The histopathological severity was evaluated by a professional pathologist as described in Methods ($n = 6$ Flox and 13 PKO, Mann-Whitney test). (**F**) Ileum histology of 9-month-old Flox and SIRT1 PKO mice after 2 cycles of DSS treatment. Representative H&E staining of Swiss swirl from 2 Flox and 2 PKO mice are shown. Bar, 1 mm. (**G**) The top enriched functional pathways in the colon or ileum of 9-month-old Flox and SIRT1 PKO mice after 2 cycles of DSS treatment. Total RNA from the indicated mice were analyzed by RNA-seq. DEGs between SIRT1 KO vs Flox were analyzed by GO biological process and top enriched pathways are shown (Permutation testing with the Benjamini–Hochberg adjusted *p*-values). Enrichment score represents $-\log_{10}$-transformed *p*-values after adjusted for FDR false discovery rate. (**H**) The small intestine of DSS-treated SIRT1 PKO mice have increased expression of immune cell genes. The expression of indicated genes was analyzed by qPCR ($n = 6$ Flox and 12 PKO mice, Student's t-test). (**I**) The expression of β-catenin was analyzed by IHC in the ileum of DSS-treated Flox and SIRT1 PKO mice. Bar, 50 µm. Data information: in (**A, B, C**, and **H**), values are expressed as mean ± SEM; in (**E**), box-and-whisker plot with the box representing the interquartile range (Q1 to Q3), a line inside indicating the median, and whiskers representing the 2.5–97.5 percentile; $^*p < 0.05$, $^{**}p < 0.01$; no marks, not significant. Source data are available online for this figure.

This is a U.S. Government work and not under copyright protection in the US; foreign copyright protection may apply

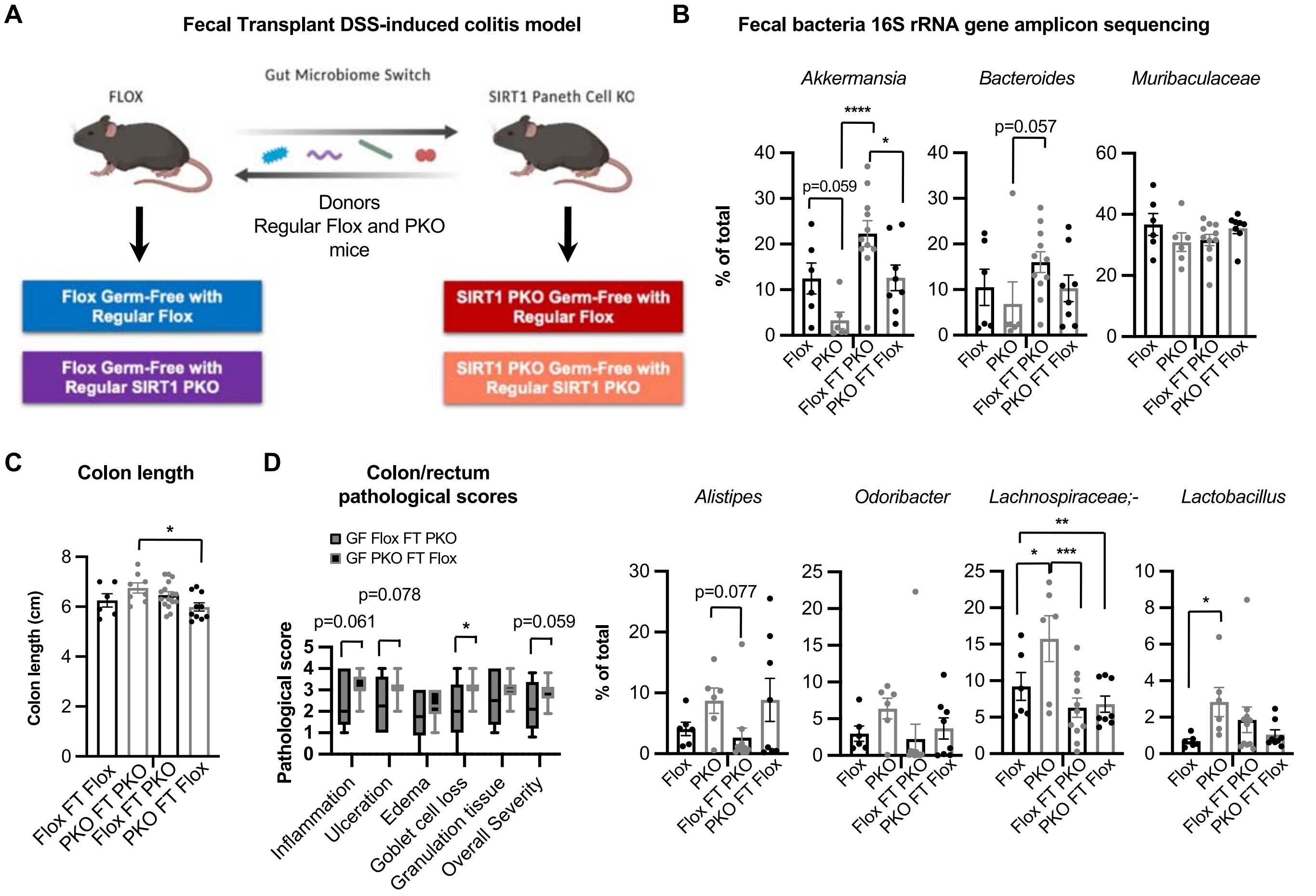

Figure EV5. Paneth cell SIRT1 regulates the sensitivity to DSS-colitis through the gut microbiota.

(**A**) Schematic representation of fecal transplantation DSS-colitis model. Germ-free Flox mice transplanted with fecal microbes from regular Flox mice (Flox FT Flox) or PKO mice (Flox FT PKO), and germ-free PKO mice transplanted with fecal microbes from regular Flox mice (PKO FT Flox) or PKO mice (PKO FT PKO) were treated with 2.5% DSS. (**B**) The abundance of major genera of fecal bacteria before and after fecal transplantation. Fecal transplantation was performed as described in Methods and fecal microbiota were analyzed by 16S rRNA amplicon sequencing ($n = 6$, 6, 11, and 8 mice, two-way ANOVA). (**C**) Colon length of DSS-treated mice (n = 6 GF Flox FT Flox, 7 GF PKO FT PKO, 16 GF Flox FT PKO, and 11 GF PKO FT Flox, two-way ANOVA). (**D**) The histopathological severity of DSS-treated mice evaluated by a professional pathologist as described in Methods ($n = 14$ GF Flox FT PKO, and 10 GF PKO FT Flox, Student's t-test). Data information: in (**B** and **C**), values are expressed as mean ± SEM; in (**D**), box-and-whisker plot with the box representing the interquartile range (Q1 to Q3), a line inside indicating the median, and whiskers representing the 2.5–97.5 percentile; *$p < 0.05$, **$p < 0.01$, ***$p < 0.001$, ****$p < 0.0001$; no marks, not significant. Source data are available online for this figure.

This is a U.S. Government work and not under copyright protection in the US; foreign copyright protection may apply
